# Iron-Induced Respiration Promotes Antibiotic Resistance in Actinomycete Bacteria

**DOI:** 10.1128/mbio.00425-22

**Published:** 2022-03-31

**Authors:** Joon-Sun Choi, Yeong-Jae Seok, You-Hee Cho, Jung-Hye Roe

**Affiliations:** a Laboratory of Molecular Microbiology, School of Biological Sciences, College of Natural Sciences and Institute of Microbiology, Seoul National University, Seoul, South Korea; b Laboratory of Microbial Physiology, School of Biological Sciences, College of Natural Sciences and Institute of Microbiology, Seoul National University, Seoul, South Korea; c Department of Pharmacy, College of Pharmacy and Institute of Pharmaceutical Sciences, CHA University, Seongnam, Gyeonggi-do, South Korea; Georgia Institute of Technology School of Biological Sciences

**Keywords:** *Streptomyces*, antibiotic resistance, bactericidal antibiotic, iron-induced respiration, actinomycetes, iron, respiration

## Abstract

The bacterial response to antibiotics eliciting resistance is one of the key challenges in global health. Despite many attempts to understand intrinsic antibiotic resistance, many of the underlying mechanisms still remain elusive. In this study, we found that iron supplementation promoted antibiotic resistance in Streptomyces coelicolor. Iron-promoted resistance occurred specifically against bactericidal antibiotics, irrespective of the primary target of antibiotics. Transcriptome profiling revealed that some genes in the central metabolism and respiration were upregulated under iron-replete conditions. Iron supported the growth of S. coelicolor even under anaerobic conditions. In the presence of potassium cyanide, which reduces aerobic respiration of cells, iron still promoted respiration and antibiotic resistance. This suggests the involvement of a KCN-insensitive type of respiration in the iron effect. This phenomenon was also observed in another actinobacterium, Mycobacterium smegmatis. Taken together, these findings provide insight into a bacterial resistance strategy that mitigates the activity of bactericidal antibiotics whose efficacy accompanies oxidative damage by switching the respiration mode.

## INTRODUCTION

Antibiotic resistance is one of the threatening problems in public health. More than 700,000 people die of infections caused by antibiotic-resistant bacteria annually (1, 2). The main mechanisms of antibiotic resistance are reduction in the drug influx, enhancement of the drug efflux, inactivation of the drug, and modification of the drug target (3–5). In addition to these quintessential resistance mechanisms that are often acquired by spontaneous mutation and/or horizontal gene transfer, recent studies suggest that the intrinsic physiological responses could affect the efficacy of not just a single antibiotic but multiple antibiotics, although they are not directly associated with the antibiotic mode of action (6, 7). For example, the stringent response involving (p)ppGpp alarmone contributes to antibiotic resistance (8, 9): inactivation of the genes for (p)ppGpp synthesis sensitizes the bacterial cells to different classes of antibiotics, allowing enhanced antibiotic efficacy in experimental infections. Production of endogenous hydrogen sulfide (H_2_S) can induce antibiotic resistance, and the chemicals that inhibit H_2_S production render the bacterial cells hypersusceptible to antibiotics (10). Moreover, alterations in the anabolic and/or catabolic pathways could also affect the efficacy of multiple antibiotics, although they are ultimately affected by the inhibitory or lethal activity of the antibiotics (11, 12). These physiological responses are regarded as the intrinsic resistance mechanisms that function irrespective of the primary targets of antibiotics and are relatively conserved among bacterial species (13).

The *Streptomyces* species are well-studied soil-dwelling bacteria famous for producing many commercially available antibiotics and other clinically important drugs (14). As vigorous antibiotic producers, streptomycetes are inevitably equipped with specialized resistance mechanisms to protect themselves specifically from endogenously produced antibiotics (15, 16). Nevertheless, the intrinsic or generalized resistance mechanisms have not been well defined in *Streptomyces* spp. except for a few examples. In Streptomyces coelicolor, a model organism for *Streptomyces* biology (17), a transcriptional regulator (WblC/WhiB7) is known to control intrinsic resistance to ribosome-targeting antibiotics similar to its role in the pathogenic actinomycete bacterium Mycobacterium tuberculosis (18, 19). It is noteworthy that the WblC/WhiB7 regulon includes not only the specialized resistance genes for drug efflux and modification but also genes for more general functions to confer resistance by modifying ribosome and translation machinery, gene transcription, and metabolism (18). Thus, more complicated resistance determinants that this antibiotic-producing bacterium exploits deserve further research to elucidate the mechanisms of intrinsic resistance to antibiotics.

Herein, we demonstrate that iron promotes the resistance of S. coelicolor to bactericidal antibiotics by changing cell metabolism. Iron is an essential nutrient for most microorganisms and is relatively rich in the soil environment (20), which implies the potential impact of iron utilization in this soil bacterium. We profiled the transcriptomic responses to antibiotics under iron-replete conditions to identify the dramatic changes in the genes for central metabolism and respiration. More importantly, iron could induce altered respiration in S. coelicolor and in Mycobacterium smegmatis as well, accounting for the intrinsic resistance of both actinomycete bacteria to the bactericidal antibiotics.

## RESULTS

### Streptomyces coelicolor displays kanamycin resistance under iron-replete conditions.

**FIG 1:**
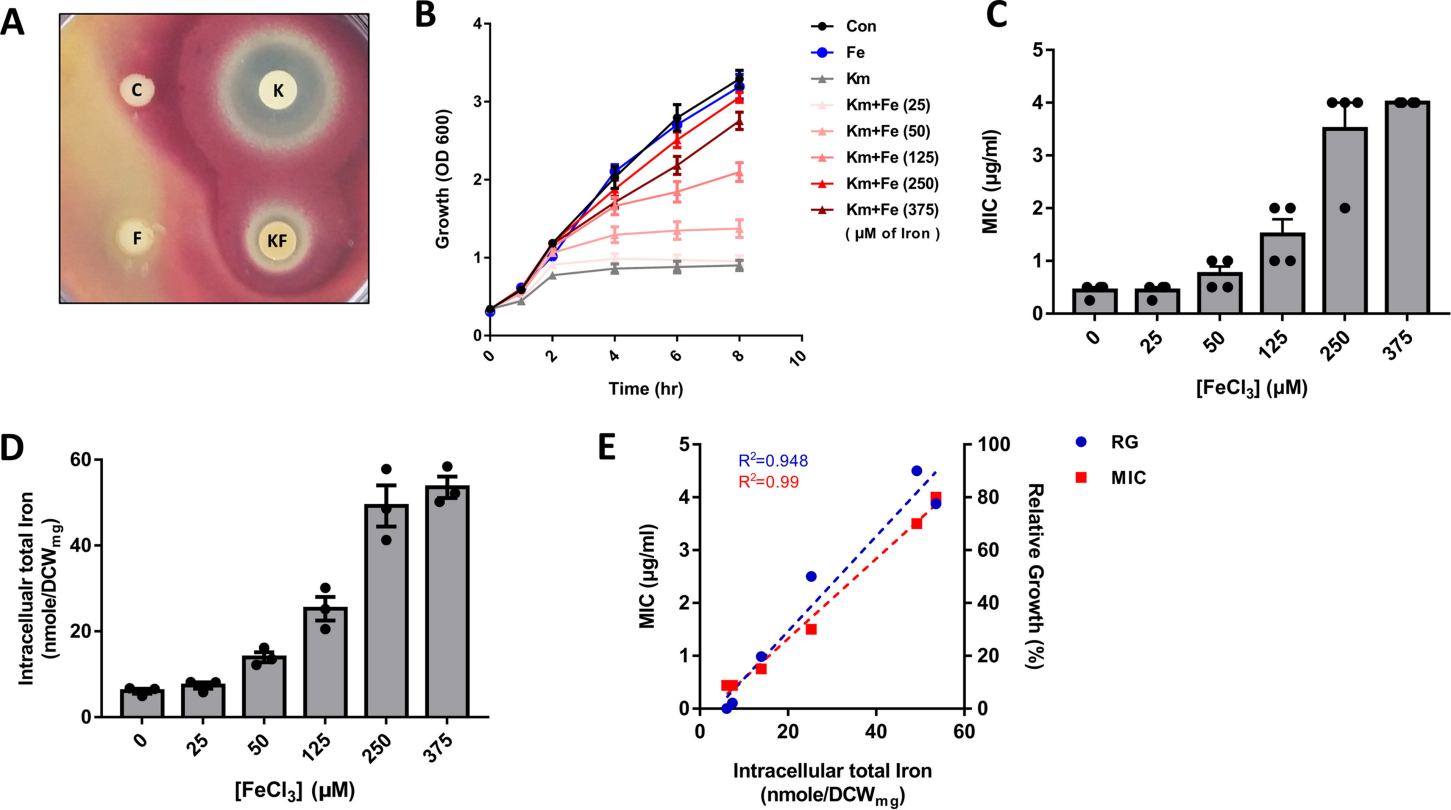
Iron induces resistance to kanamycin. (A) Disk diffusion assay for kanamycin sensitivity under iron-replete condition. Labels: C, no treatment; K, kanamycin (25 μg); F, FeCl_3_ (15 μg); KF, kanamycin and iron. (B and C) Growth curves (B) and MIC values (C) in the presence of kanamycin (Km; 0.5 μg/mL) and iron (Fe) at the iron concentrations (μM) indicated in parentheses. Con, no treatment (black circles). (D) Intracellular total iron contents were measured after iron supplementation at the designated concentrations in panels B and C. Iron contents were normalized by dry cell weight (nmole/DCW_mg_). (E) Correlation between intracellular iron content from panel D and either MIC of kanamycin (red squares) or relative growth (RG; blue circles) from panels B and C. RG (%) was calculated using the OD_600_ values at 8 h in panel B. *R*^2^ values were calculated from linear regression.

During our research on superoxide dismutases (SODs) in S. coelicolor, we fortuitously observed that the activity of iron-containing SOD (SodF) was specifically reduced in the presence of a sublethal level of kanamycin (0.25 μg/mL) (see Fig. S1 in the supplemental material). Based on the possible link between the growth inhibitory effect of kanamycin and the SodF depletion that is usually caused by reduced iron availability as an iron-sparing response (21), we hypothesized that kanamycin-induced growth inhibition might be attributed to iron starvation. To test for this, we examined whether the bacterial susceptibility to kanamycin could be circumvented simply by iron repletion. As shown in Fig. 1A, the result from a disk diffusion assay showed that the zone of inhibition by kanamycin was significantly reduced by iron supplementation. This was also verified by measuring the growth and determining the MICs of kanamycin in the presence of iron. As shown in Fig. 1B, growth inhibition by kanamycin gradually disappeared as more iron was supplemented in liquid culture, whereas growth was not affected at all by the iron treatment in the absence of kanamycin. The MIC of kanamycin was concomitantly increased with iron treatment in a dose-dependent manner (Fig. 1C). The intracellular iron levels were increased in proportion to the concentrations of supplied iron (Fig. 1D), as measured by a ferrozine-based spectrophotometric assay. In the presence of kanamycin, the relative growth and MIC values correlated very well with the intracellular iron levels (Fig. 1E). These results led us to conclude that iron treatment promotes kanamycin resistance in S. coelicolor. In contrast to the effect of iron, other transition metals such as cobalt, nickel, manganese, and zinc were unable to induce kanamycin resistance (Fig. S2A and B), suggesting that some iron-specific physiology could obviate the antibiotic effect exerted by kanamycin.

10.1128/mbio.00425-22.1 FIG S1 Kanamycin treatment and superoxide dismutase activity. Native PAGE was performed for activity staining using 20 μg of cell lysates from S. coelicolor that had been treated for the indicated time (0.5, 1, and 2 h) with (+) or without (−) 1 μg/mL kanamycin (Km). The activity bands of Ni-containing (SodN) and Fe-containing (SodF) SODs are indicated on the left. Download FIG S1, PDF file, 0.1 MB. Copyright © 2022 Choi et al. 2022 Choi et al. https://creativecommons.org/licenses/by/4.0/ This content is distributed under the terms of the Creative Commons Attribution 4.0 International license.

10.1128/mbio.00425-22.2 FIG S2 Specificity of iron in promoting kanamycin resistance. (A) Relative growth (%) was calculated using the OD_600_ values after 8 h of treatment with 0.5 μg/mL kanamycin in the absence or presence of various metals (Fe, FeCl_3_; Co, CoCl_2_; Ni, NiSO_4_; Mn, MnCl_2_; Zn, ZnCl_2_) at 250 μM. (B) MIC values of kanamycin in the presence of various metals at the designated concentrations. The values shown are the means, with error bars representing the standard deviations, of results from three independent experiments. The *P* value was indicated from a two-way ANOVA with Dunnett’s multiple-comparison test. Download FIG S2, PDF file, 0.2 MB. Copyright © 2022 Choi et al. 2022 Choi et al. https://creativecommons.org/licenses/by/4.0/ This content is distributed under the terms of the Creative Commons Attribution 4.0 International license.

### Resistance to polymyxin B and other aminoglycosides is also enhanced by iron.

**FIG 2:**
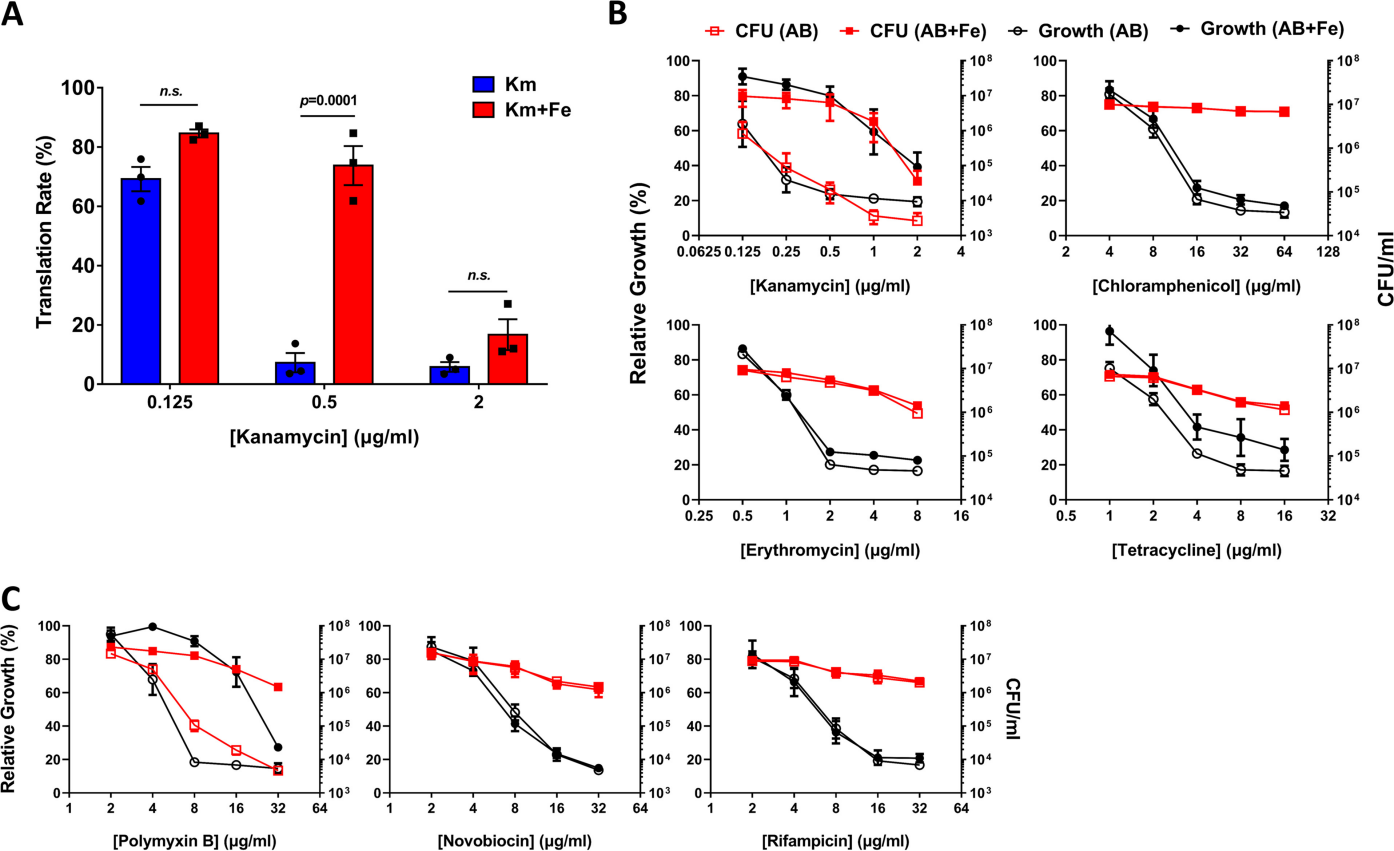
Iron induces resistance to kanamycin and polymyxin B. (A) Translation rate (%) calculated from [S^35^]Met incorporation under kanamycin only (blue; Km) or kanamycin plus iron (red; Km+Fe). The values shown are the means, with error bars representing the standard deviation, of results from three independent experiments. *P* value is indicated from two-way analysis of variance (ANOVA) with Sidak’s multiple-comparison test. n.s., nonsignificant. (B and C) Relative growth (black circles; left axis) and number of CFU (red squares; right axis) of S. coelicolor cells cultured in the presence of ribosome-targeting antibiotics (B) or other antibiotics (C) at designated concentrations with (AB+Fe,; filled symbols) or without (AB; open symbols) iron are shown.

Since kanamycin is one of the well-studied ribosome inhibitors (22), we investigated whether iron (250 μM) could restore the ribosome-mediated protein synthesis rate based on [^35^S]Met incorporation. As shown in Fig. 2A, iron supplementation could alleviate the translational dysfunction caused by kanamycin (0.5 μg/mL). One can postulate that iron may have altered the ribosomes to become more resistant to kanamycin, considering that iron could replace magnesium, an essential cofactor in the ribosome (23). If this was the case, iron enhancement of antibiotic resistance would be observed against other ribosome inhibitors. In order to test this idea, we measured the effect of iron on growth (optical density [OD]) and cell viability (number of CFU) in the presence of chloramphenicol, erythromycin, and tetracycline. Figure 2B shows that iron did not induce resistance against other ribosome inhibitors. Therefore, general alteration of ribosomes by iron to resist translational inhibitors could not be the plausible mechanism behind the iron effect.

We then examined the effect of iron on the efficacies of other antibiotics that target the cell envelope (polymyxin B), DNA replication (novobiocin), or transcription (rifampin). Interestingly, iron-induced resistance was observed only against polymyxin B (Fig. 2C), which interferes with the membrane integrity of the Gram-negative bacteria and a few Gram-positive bacteria by direct interaction with some negatively charged components of the cell envelope (24). We first ruled out the possibility that kanamycin and polymyxin B could directly affect iron availability by determining the intracellular iron levels and the expression levels of the iron-responsive genes (Fig. S3). A common feature of kanamycin and polymyxin B that distinguishes them from the other antibiotics is that both of them are bactericidal, whereas the others are bacteriostatic (Fig. 2B and C). We then examined whether iron promotes resistance to other bactericidal aminoglycosides, such as gentamicin, streptomycin, and neomycin. The results demonstrated that iron enhanced the resistance to all these aminoglycosides (Fig. S4). Therefore, we propose that iron could induce resistance to bactericidal antibiotics such as aminoglycosides and polymyxin B, regardless of the direct target points of the antibiotics.

10.1128/mbio.00425-22.3 FIG S3 Antibiotic treatment and intracellular iron status. (A) Intracellular total iron contents were measured before (control) and after treatments with 0.5 μg/mL kanamycin (Km; red) or 8 μg/mL polymyxin B (PMX; blue) for 30 min. Measured values were normalized by dry cell weight (DCW_mg_). (B) Expression levels of genes for iron regulator (*dmdR*), DmdR regulon (*desA, cchE, sco7400*), or iron-containing enzyme (*sodF*) were measured after treatment with 100 μM dipyridyl (Dip; black), 0.5 μg/mL kanamycin (Km; red), or 8 μg/mL polymyxin B (PMX; blue) for 30 min. Gene expression was quantified by using reverse transcription-quantitative PCR (qRT-PCR), and fold induction was calculated relative to the nontreated control. The values shown are the means, with error bars representing the standard deviations, of results from three independent experiments. Download FIG S3, PDF file, 0.1 MB. Copyright © 2022 Choi et al. 2022 Choi et al. https://creativecommons.org/licenses/by/4.0/ This content is distributed under the terms of the Creative Commons Attribution 4.0 International license.

10.1128/mbio.00425-22.4 FIG S4 Iron promotes resistance to aminoglycoside drugs. Relative growth (black circles; left axis) and CFUs (red squares; right axis) of S. coelicolor cells cultured in the presence of gentamicin, streptomycin, or neomycin at the designated concentrations with (AB+Fe; filled symbols) or without (AB; open symbols) iron are shown. Download FIG S4, PDF file, 0.1 MB. Copyright © 2022 Choi et al. 2022 Choi et al. https://creativecommons.org/licenses/by/4.0/ This content is distributed under the terms of the Creative Commons Attribution 4.0 International license.

### Iron enhances the respiration of S. coelicolor.

**FIG 3:**
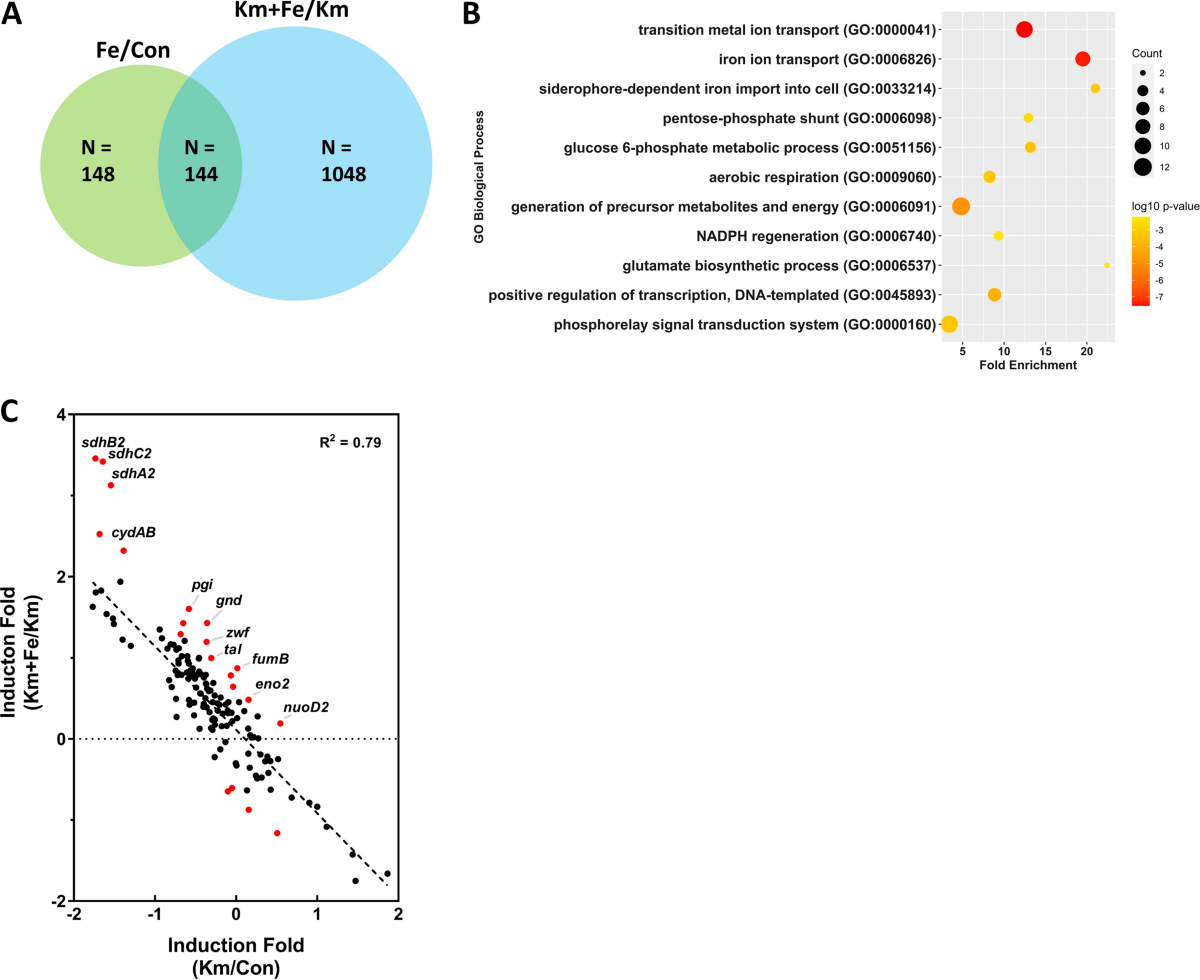
Transcriptomic analysis of iron-supplemented S. coelicolor. (A) Venn diagram with the numbers of iron-responsive genes. Iron-responsive genes were selected based on their adjusted P values of less than 0.05. (B) Dot plot for iron-responsive genes based on the gene ontology (GO) terms. The size of the dots represents the counted number of genes in each ontology term, and the color of the dots represents the significance of each ontology enrichment using the *P* value (−log_10_ *P*; from yellow to red). (C) Scatterplot for the 144 genes by kanamycin-stressed (Km/Con) and iron-treated (Km+Fe/Km) conditions. The *R*^2^ value was calculated from linear regression. Genes with a difference in fold induction of >5 are marked in red, and those belonging to carbon metabolism and respiration are marked with the gene names.

As an initial attempt to elucidate the mechanisms by which iron could promote antibiotic resistance, we profiled transcriptome changes by RNA sequencing upon iron supplementation in the presence and absence of antibiotic treatment (Fig. 3). Iron changed significantly more genes in the presence of kanamycin than in its absence. Among the 1,192 genes whose expression was changed significantly by iron in the presence of kanamycin, the expression of 1,048 genes was mostly changed to levels that were similar to the untreated (control) levels. The principal-component analysis (PCA) plot reflects this phenomenon by showing that iron shifts the clustering pattern of the kanamycin-treated transcriptome toward the transcriptome pattern of the no-kanamycin sample (Fig. S5). We therefore further analyzed the 144 genes whose expression level changed by iron treatment in the presence of kanamycin as well as in its absence (Fig. 3A and Table S2). Except for iron transporter genes, those belonging to the functional groups for central carbon metabolism (*pgi*, *gnd*, *zwf*, *tal*, *fumB*, and *sdh2* operon) and respiration (*cydAB* and *sdh2* operon) were highly enriched in this list (Fig. 3B and C). To examine whether these genes are required for iron-promoted antibiotic resistance, we created deletion strains that lack the *gnd-pgi-zwf-tal* operon (SCO6658-6663), *sdhB2A2C2* operon (SCO0922-0924), or *cydAB* operon (SCO3945-3946) or an overexpression strain of the *fumB* gene (SCO5044) driven by the ermE* promoter. When we measured the number of CFU of each strain in the presence of kanamycin, we found that the mutations or overexpression did not compromise the iron effect (Fig. S6). However, the negative results still do not exclude the possibility that these genes contribute to iron-promoted antibiotic resistance, since the effect of single mutations can often be compensated by other alternative genes or pathways.

10.1128/mbio.00425-22.5 FIG S5 Principal-component analysis (PCA) of differential expression data sets from S. coelicolor. PCA plot of RNA-seq data sets for the gene expression changes in S. coelicolor cells that were untreated (red; control) or treated with 250 μM FeCl_3_ (yellow), 0.5 μg/mL kanamycin (green), or 0.5 μg/mL kanamycin plus 250 μM FeCl_3_ (purple). Each data set consisted of two independent transcriptome data (spot). The PCA was performed by using normalized RNA-seq data of 8,152 genes differentially expressed in at least one pairwise comparison: control versus iron and/or kanamycin treatment. Download FIG S5, PDF file, 0.1 MB. Copyright © 2022 Choi et al. 2022 Choi et al. https://creativecommons.org/licenses/by/4.0/ This content is distributed under the terms of the Creative Commons Attribution 4.0 International license.

10.1128/mbio.00425-22.6 FIG S6 Effect of deletion or overexpression of some metabolic genes on iron-promoted kanamycin resistance. CFU/mL was enumerated by counting viable cells of wild-type, deletion mutant, or overexpression strains after 8 h of culture in the presence of kanamycin (0.5 μg/mL) treatment with (Fe+) or without (none) FeCl_3_ (250 μM) supplementation. The examined strains are mutants with deletions of SCO4855-4858 (Δ*sdh* complex), SCO0922-0924 (Δ*sdh2* complex), SCO6658-6663 (Δ*gnd*-*tktB* operon), and SCO3945-3946 (Δ*cydAB*) and an overexpression strain of SCO5044 driven by the ermE* promoter (ermE*::*fumB*). The values shown are the means, with error bars representing the standard deviations, of results from three independent experiments. Download FIG S6, PDF file, 0.1 MB. Copyright © 2022 Choi et al. 2022 Choi et al. https://creativecommons.org/licenses/by/4.0/ This content is distributed under the terms of the Creative Commons Attribution 4.0 International license.

10.1128/mbio.00425-22.10 TABLE S2 List of 144 genes responsive to iron. Download Table S2, PDF file, 0.1 MB. Copyright © 2022 Choi et al. 2022 Choi et al. https://creativecommons.org/licenses/by/4.0/ This content is distributed under the terms of the Creative Commons Attribution 4.0 International license.

**FIG 4:**
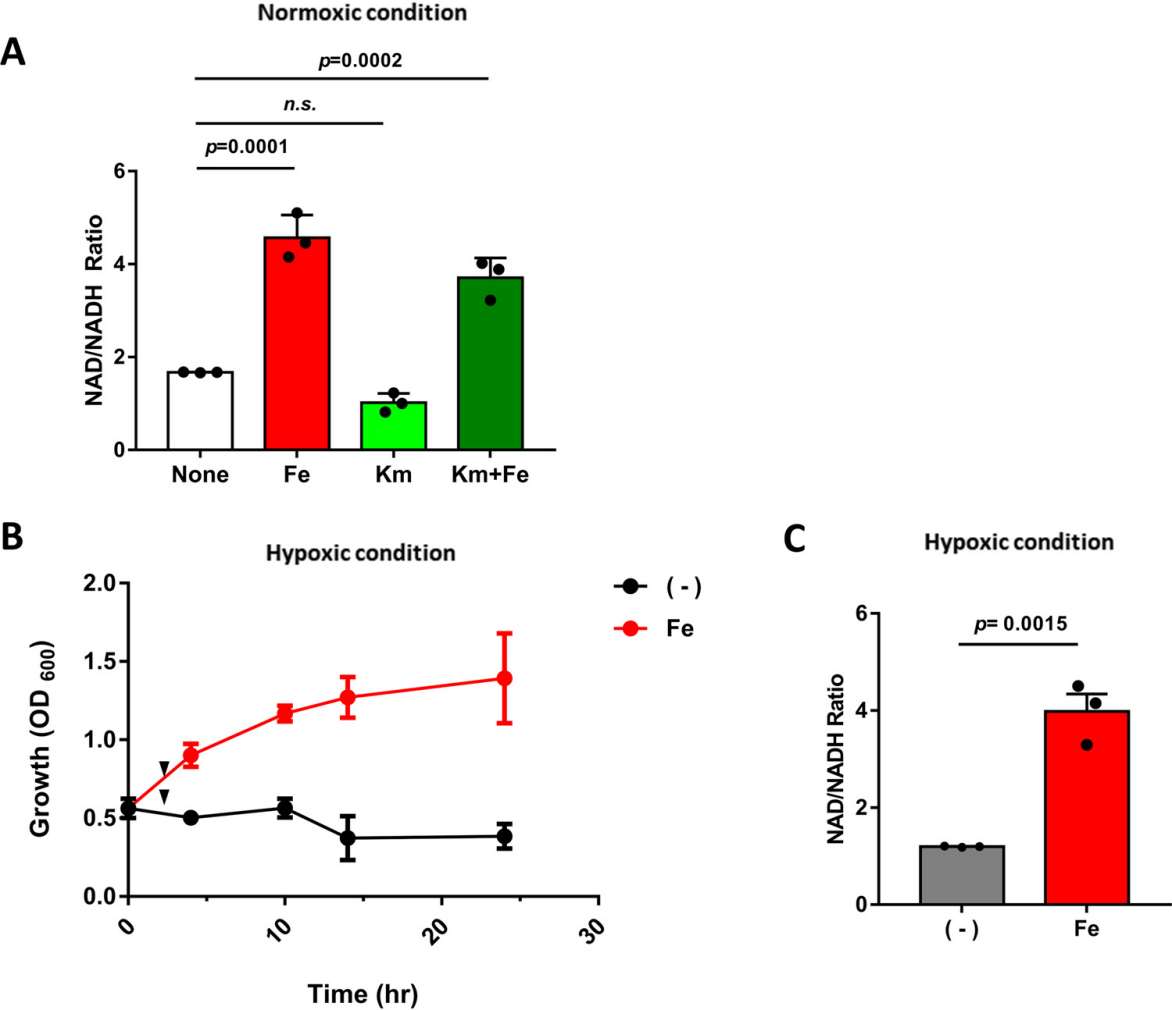
Iron-induced respiration under hypoxic condition. (A) Quantification of the intracellular amount of NAD and NADH after treatment with 0.5 μg/mL kanamycin (Km) and/or 250 μM iron (Fe) for 30 min. Measurements were normalized by dry cell weight (DCW_mg_). (B) Growth (OD_600_) of S. coelicolor with (Fe; red) or without (−; black) iron treatment under hypoxic conditions in an anaerobic chamber. Arrowheads indicate the time point (120 min) of NAD/NADH measurement in panel C. (C) Quantification of the intracellular ratio of NAD to NADH during the microaerophilic growth in panel B. The amounts of NAD and NADH were measured, and the ratio was normalized by dry cell weight (DCW_mg_). The values shown are the means, with error bars representing the standard deviations, of results from three independent experiments. The *P* value is indicated from one-way ANOVA with Dunnett’s multiple-comparison test (A) and a two-tailed *t* test (C). n.s., nonsignificant.

It was noted that both the *cydA* and *cydB* genes encoding two subunits of cytochrome *bd* oxidase were upregulated upon iron supplementation in kanamycin treatment (Fig. 3C). Cytochrome *bd* oxidase is an alternative terminal oxidase that is induced by oxygen limitation in S. coelicolor as well as in other bacteria (25, 26). This observation and the previous findings that the toxicity of bactericidal antibiotics is derived from aerobic respiration (27) led us to hypothesize that iron-promoted resistance to the two bactericidal antibiotics may be associated with some alteration in respiration in S. coelicolor. To test this idea, we measured the amount of intracellular NAD^+^ and NADH and calculated their ratio as an indicator of respiration activity. As shown in Fig. 4A, iron supplementation increased NADH oxidation whereas kanamycin decreased it. Iron could restore the NADH oxidation that was affected by kanamycin up to a level of about 2-fold of the untreated control level. This result is consistent with the aforementioned data on the growth-stimulating effect of iron in the presence of kanamycin (Fig. 1B and 2B). The intracellular ATP levels were not significantly changed by iron and/or kanamycin treatment (Fig. S7). There is a possibility that the proton motive force or ATP generated by iron-promoted NADH oxidation could have been consumed by other cellular processes such as membrane transport. Overall, our data suggest that the growth-stimulatory effect of iron in the presence of bactericidal antibiotics seems to result from altered respiration.

10.1128/mbio.00425-22.7 FIG S7 Levels of ATP under kanamycin and iron treatments. Quantification of the intracellular amount of ATP after treatments with 0.5 μg/mL kanamycin (Km) and/or 250 μM iron (Fe) for 30 min. Measurements were normalized by dry cell weight (DCW_mg_). The values shown are the means, with error bars representing the standard deviations, of results from three independent experiments. Download FIG S7, PDF file, 0.09 MB. Copyright © 2022 Choi et al. 2022 Choi et al. https://creativecommons.org/licenses/by/4.0/ This content is distributed under the terms of the Creative Commons Attribution 4.0 International license.

Considering the function of CydAB as an alternative oxidase under oxygen-limiting conditions and its upregulation upon iron treatment, it can be predicted that iron can stimulate bacterial growth under oxygen-limiting conditions. To test this idea, we monitored the growth of S. coelicolor, known to be a strictly aerobic organism, in an anaerobic chamber in the presence or absence of iron. As shown in Fig. 4B, the growth of S. coelicolor was severely compromised under this condition, as expected. However, its growth was significantly stimulated by iron supplementation in the anaerobic chamber. Iron supplementation increased NADH oxidation under anaerobic conditions, as shown in Fig. 4C. Accumulation of NADH to a level equivalent to that of NAD^+^ (i.e., NAD^+^/NADH ratio of ∼1.0) appeared to coincide with the growth defect of S. coelicolor under anaerobic conditions. Iron enhanced this ratio to ∼4.0. These observations support the idea that iron alters respiration to a mode(s) that is less dependent on oxygen and hence enhances bacterial resistance to bactericidal antibiotics, whose efficacy is closely linked to oxygen-dependent respiration (27).

### KCN-mediated respiration impairment reduces but not abolishes iron-promoted antibiotic resistance.

**FIG 5:**
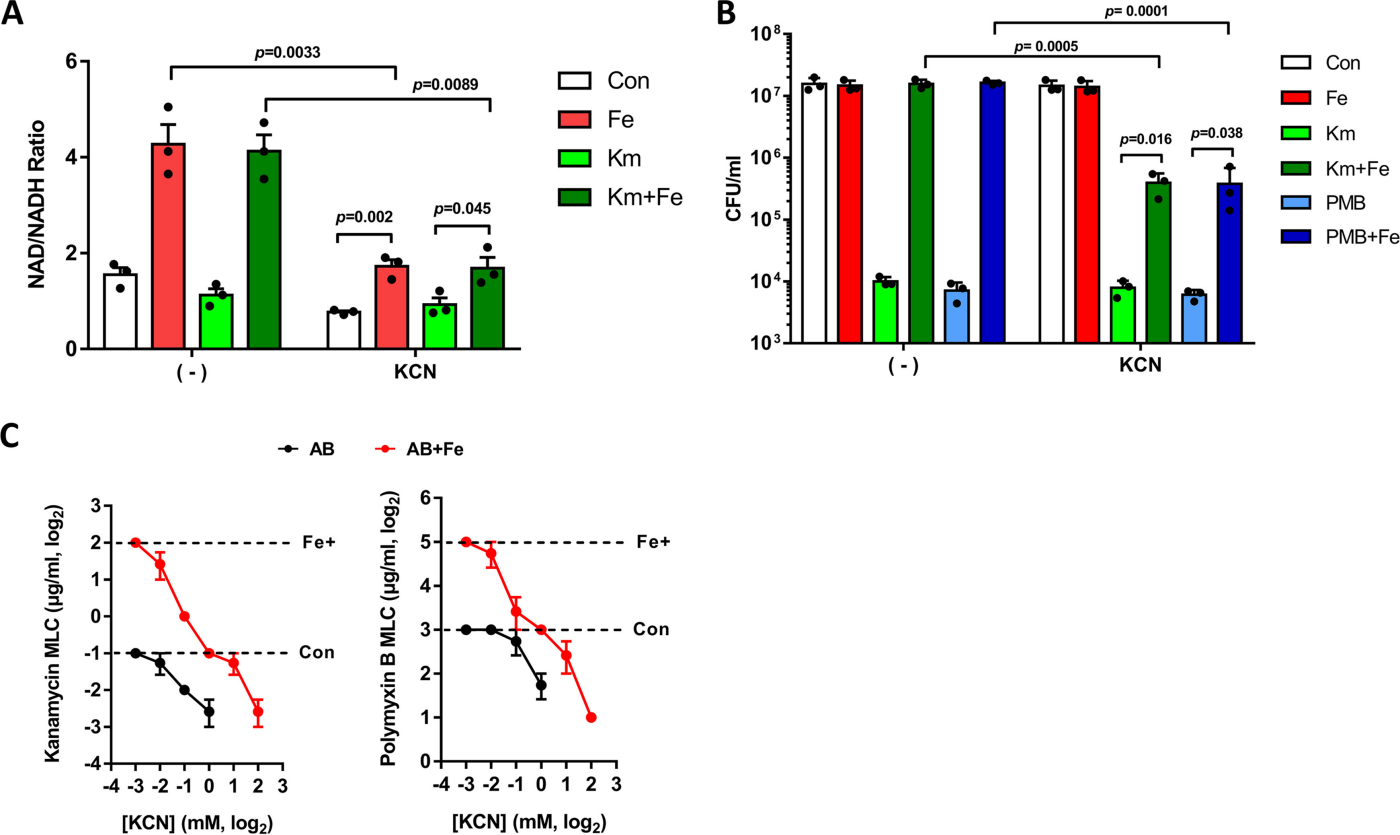
KCN treatment and antibiotic resistance under iron-replete conditions. (A) Ratio of NAD to NADH as shown in Fig. 4A after KCN treatment (0.5 mM) for 30 min in the presence of kanamycin and iron (Km+Fe). Treatment with either kanamycin (Km, 0.5 μg/mL) or iron (Fe, 250 μM) was included with a no-treatment control (Con). (B) Numbers of CFU as shown in Fig. 2B after KCN treatment for 30 min. CFU/mL were enumerated by viable counts of serially diluted 8-h cultures in the presence of antibiotics and iron (Km+Fe and PMB+Fe). PMB, polymyxin B. Treatment with kanamycin (Km, 0.5 μg/mL), PMB (8 μg/mL), or iron (Fe, 250 μM) was included with a no-treatment control (Con). (C) MLC values of kanamycin or PMB by KCN (∼0.125 to 4 mM) treatment for 30 min. Each MLC value was determined with (AB+Fe) or without (AB; black) iron. The values shown are the means, with error bars representing the standard deviations, of results from three independent experiments. *P* values are indicated from an unpaired multiple *t* test. n.s., nonsignificant.

To further verify the contribution of altered respiration to iron-promoted antibiotic resistance, we examined the effect of potassium cyanide (KCN), a well-known inhibitor of terminal oxidases in aerobic respiration (28). As shown in Fig. 5A, KCN at its sublethal concentration (0.5 mM) reduced respiration in all samples by about 2-fold. However, even in the presence of KCN, iron still promoted respiration significantly, suggesting the involvement of a KCN-insensitive type of respiration in the iron effect. We then examined the effect of KCN on the viability of cells treated with bactericidal antibiotics (kanamycin and polymyxin B) in the presence and absence of iron. Figure 5B shows that iron-promoted survival against bactericidal antibiotics (kanamycin and polymyxin B) was decreased under 0.5 mM KCN, as assessed by measuring the number of CFU. However, iron still enhanced survival against both antibiotics significantly in the presence of KCN, coinciding with the results shown in Fig. 5A. We further examined the effect of KCN on iron-promoted antibiotic resistance at different concentrations (∼0.125 to 4 mM) by determining minimal lethal concentrations (MLCs) of kanamycin and polymyxin B. Results in Fig. 5C demonstrate that the MLCs of kanamycin and polymyxin B were decreased by KCN treatment, but under all concentrations tested, MLCs were higher in the presence of iron. KCN did not affect the MICs of other bacteriostatic antibiotics in the presence or absence of iron as much as it did for bactericidal antibiotics (Fig. S8). Although iron-altered respiration and iron-promoted resistance were both affected by 0.5 mM KCN (Fig. 5A and B), it is noteworthy that there still remained the iron-promoting effect, insensitive to 0.5 mM KCN, that enhanced cell viability against bactericidal antibiotics (Fig. 5B). This implies that the iron-altered respiration involves at least two different systems: one that is susceptible to KCN and the other that is relatively insensitive to KCN at its sublethal concentration. Taking all these data together, we propose that iron-promoted resistance to bactericidal antibiotics can be attributed to an altered respiration mode in S. coelicolor, a large portion of which is relatively insensitive to KCN.

10.1128/mbio.00425-22.8 FIG S8 Effect of KCN on MICs of bacteriostatic antibiotics in the presence or absence of iron supplementation. MIC values of bacteriostatic antibiotics by KCN treatment for 30 min. Each MIC value was determined with (AB+Fe; red) or without (AB; black) iron. The values shown are the means, with error bars representing the standard deviations, of results from three independent experiments. Download FIG S8, PDF file, 0.1 MB. Copyright © 2022 Choi et al. 2022 Choi et al. https://creativecommons.org/licenses/by/4.0/ This content is distributed under the terms of the Creative Commons Attribution 4.0 International license.

### Mycobacterium smegmatis also displays antibiotic resistance under iron-replete conditions.

**FIG 6:**
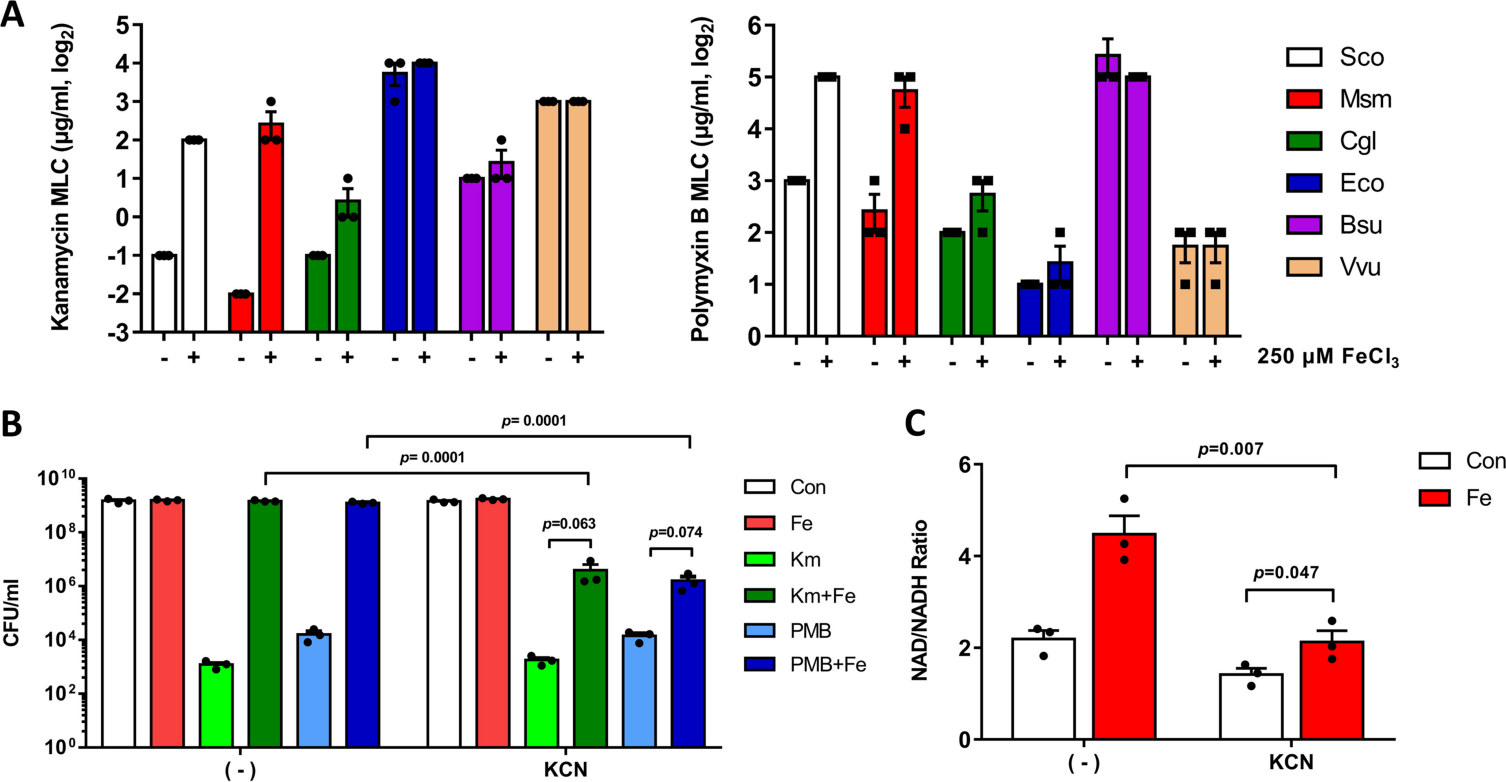
Iron-promoted antibacterial resistance is conserved in Mycobacterium smegmatis. (A) MLC values of kanamycin and PMB in bacterial species (Sco, S. coelicolor; Msm, M. smegmatis; Cgl, C. glutamicum; Eco, E. coli; Bsu, B. subtilis; Vvu, V. vulnificus) in the presence (+) or absence (−) of iron. (B) CFU as shown in Fig. 5B by KCN treatment for 30 min. CFU/mL were enumerated by viable counts of serially diluted 12-h cultures in the presence of antibiotics and iron (Km+Fe and PMB+Fe) in M. smegmatis. Treatment with kanamycin (Km), PMB, or iron (Fe) was included with a no-treatment control (Con). (C) Ratio of NAD to NADH after KCN treatment for 30 min in the presence (Fe) or absence (Con) of iron in M. smegmatis. The values shown are the means, with error bars representing the standard deviations, of results from three independent experiments. *P* values are indicated (unpaired multiple *t* test). n.s., nonsignificant.

We next investigated whether this iron effect is unique to S. coelicolor or streptomycetes, since the bacteria of the genus *Streptomyces* are producers of diverse antibiotics in nature (14). The effect of iron on resistance to kanamycin and polymyxin B was examined in other bacteria from the phyla *Actinobacteria* (M. smegmatis and Corynebacterium glutamicum), *Firmicutes* (Bacillus subtilis), and *Proteobacteria* (Escherichia coli and Vibrio vulnificus). As shown in Fig. 6A, we observed that M. smegmatis and, to a lesser extent, C. glutamicum displayed iron-promoted resistance to both of the bactericidal antibiotics, kanamycin and polymyxin B. The effect of KCN on number of CFU and NADH oxidation was examined in M. smegmatis as shown in Fig. 5. As demonstrated in Fig. 6B and C, treatment with a sublethal concentration of KCN led to reductions in both CFU and NADH oxidation under iron-replete conditions, as observed in S. coelicolor. It was also similarly observed that KCN did not completely reduce the NADH oxidation and number of CFU in M. smegmatis, implying the existence of a KCN-insensitive portion in iron-altered respiration (Fig. 6B and C). Therefore, the iron-promoted resistance to bactericidal antibiotics appears to be a conserved phenomenon in actinobacteria and occurs most likely through the alteration of respiration.

## DISCUSSION

In this study, we demonstrated that increased intracellular iron promotes intrinsic antibiotic resistance through altered respiration in two actinobacterial species, S. coelicolor and M. smegmatis. Actinomycetes are generally aerobic, Gram-positive bacteria and are predominantly found in soil. Since those deeper areas in soil are deemed short of oxygen and thus hypoxic (29), an alternative or microaerophilic respiration mode might be necessary for those aerobic bacteria to survive limited oxygen availability, as previously suggested for S. coelicolor (30). Oxygen is also thought to be limited near the center of the densely grown mycelial network. Moreover, iron metabolism might be also important for those bacteria thriving in soil, because soil is highly rich in iron compared to other natural habitats (20). For S. coelicolor and other soil actinomycetes that have well been adapted to such iron-rich hypoxic soil environments, iron-adapted respiration might be one of those strategies for survival. Although the precise modes by which S. coelicolor supports vegetative growth under hypoxic and iron-replete conditions need to be determined, our study makes it clear that the iron-induced alteration of respiration to enhance NADH oxidation lies behind the promotion of intrinsic resistance to bactericidal antibiotics in actinobacteria such as S. coelicolor and M. smegmatis.

The effect of KCN on reducing the magnitude of NADH oxidation and cell survival under bactericidal antibiotics suggests that the altered respiration induced by iron could consist of multiple changes in respiratory pathways for NADH oxidation, differing in KCN sensitivity. The altered respiration might involve cytochrome *bd* oxidase produced from the *cydAB* operon upregulated by iron. Although our data showed that *cydAB* mutation did not change the iron effect in S. coelicolor (see Fig. S6 in the supplemental material), there still remains a possibility that the cytochrome *bd* oxidase may play some role, considering that mutations sometimes accompany compensating changes to compromise their effects. It was previously reported that the *cydA* mutant of M. smegmatis was highly sensitive to hydrogen peroxide and the antileprotic antibiotic clofazimine (31), corroborating the potential contribution of cytochrome *bd* oxidase and possibly some other alternative oxidases (26) to the intrinsic antibiotic resistance in these bacterial species.

It should also be noted that this iron-promoted antibiotic resistance through altered respiration occurs against bactericidal antibiotics that are effective on actinobacteria, regardless of the primary targets of antibiotics. Despite some controversy about the exact nature of the culprit molecules, it has been shown that the killing effect of bactericidal antibiotics relies on the generation of reactive oxygen species (ROS) during aerobic respiration (27, 32). Recent studies also demonstrated that aerobic respiration makes the growing bacteria more susceptible to bactericidal antibiotics and facilitates eradication of persisters induced by the antibiotics (33, 34). A recent report revealed that an anaerobic respiration mode using nitrate as the final electron acceptor promotes intrinsic resistance to polymyxin B in Pseudomonas aeruginosa (35). This might be able to obviate the ROS generation that occurs during oxygen respiration in this bacterium. Likewise, it can be postulated that S. coelicolor and M. smegmatis, both of which are known as strict aerobic bacteria (36), could support the anaerobic type of respiration in an iron-rich soil environment. It has been demonstrated that S. coelicolor can utilize nitrate as an electron acceptor (37). However, nitrate respiration did not allow growth under anaerobic conditions but only maintained proton motive force enough to support dormancy, such as in spores (30). It has been postulated that alternative electron acceptors may be involved in generating proton motive force under hypoxic or anaerobic conditions, especially considering the more than 200 uncharacterized oxidoreductases in the S. coelicolor genome (17, 37). In this study, we have shown that iron can enhance respiration and hence the growth of S. coelicolor even under anaerobic conditions (Fig. 4C). How iron supports anaerobic respiration and growth of S. coelicolor and whether this phenomenon can be observed in other actinomycetes known to be strictly aerobic are intriguing questions to solve in the future.

Although iron is an essential element for growth and survival for most microorganisms, high levels of free iron may result in progressive cell damage via the Fenton reaction, which generates hydroxyl radicals by one-electron transfer to hydrogen peroxides spontaneously produced during aerobic respiration. This iron-mediated toxicity has been exploited as an antibiotic adjuvant or inhibitor (38), as reported in some studies showing that iron could enhance antibiotic-dependent cell death and that decreasing iron concentrations led to increased antibiotic resistance (39–41). In contrast, some studies reported the opposite effect of iron, such that the efficacy of antibiotics was enhanced by iron chelators (42) and that an increased iron supply induced resistance to antibiotics (43). Considering the nature of iron as a two-edged sword, this apparent discrepancy might be associated with the differential availabilities and biological accessibilities of iron that depend on the microorganisms and/or the growth conditions. However, considering that kanamycin is one of the second-line antituberculosis (anti-TB) drugs for the treatment of extensively drug-resistant TB (44), some precaution may be needed in the use kanamycin or related bactericidal antibiotics for TB patients, whose lungs usually contain more iron than those of healthy individuals (45). Further studies are necessary to reveal the detailed biochemical mechanism of iron-promoted resistance, which may provide a deeper understanding of the various intrinsic resistance mechanisms in the microorganisms.

## MATERIALS AND METHODS

### Strains, growth conditions, and reagents.

Bacterial strains used in this study are listed in Table S1A in the supplemental material. S. coelicolor strains were grown and maintained by a standard protocol (46). Proper culture media (S. coelicolor in YEME with 5 mM MgCl_2_, 10% sucrose; E. coli, B. subtilis, and C. glutamicum in LB broth; V. vulnificus in LB with 2.5% NaCl; M. smegmatis in 7H9 medium without ferric ammonium citrate and supplemented with 0.05% Tween 80, 0.2% glucose, 0.5% bovine serum albumin, 0.085% NaCl) were used. For growth of S. coelicolor under anaerobic conditions, S. coelicolor spores were germinated and grown to mid-exponential phase (OD at 600 nm [OD_600_] of ∼2.0) in YEME medium and then diluted 4-fold into YEME medium that had been placed in an anaerobic chamber (Coy) after being autoclaved overnight, followed by cultivation at 30°C under hypoxic condition.

10.1128/mbio.00425-22.9 TABLE S1 List of strains, plasmids, and primers used in this study. (A) Strains and plasmids. Am^R^, apramycin resistant. (B) Primers. F and R refer to the forward and reverse of the coding region, respectively; UP and DW refer to the upstream and downstream of the flanking regions for each gene, respectively. Download Table S1, PDF file, 0.07 MB. Copyright © 2022 Choi et al. 2022 Choi et al. https://creativecommons.org/licenses/by/4.0/ This content is distributed under the terms of the Creative Commons Attribution 4.0 International license.

For antibiotic treatment, freshly made solutions of antibiotics (kanamycin disulfate salt, tetracycline hydrochloride, chloramphenicol, erythromycin A dihydrate, fusidic acid sodium salt, polymyxin B sulfate, novobiocin sodium salt, rifampin; all from Sigma) at the indicated concentrations were applied to the early exponential-phase cells (OD_600_ of ∼0.3).

### SOD activity staining.

Superoxide dismutase (SOD) activity staining was conducted as described previously with slight modification (47). Cell lysates (20 μg) from wild-type S. coelicolor were separated on a 10% nondenaturing polyacrylamide gel. The gel was washed with distilled water and soaked in 20 mM potassium phosphate (pH 7.8) containing 30 μM riboflavin and 30 mM tetramethylethylenediamine for 30 min in the dark. The gel was transferred to 20 mM potassium phosphate (pH 7.8) containing 2.5 mM nitroblue tetrazolium for 20 min in the dark. The gel was illuminated with white light until an SOD activity band appeared.

### Gene deletion and overexpression.

Streptomyces coelicolor deletion mutants were constructed by homologous recombination. In brief, flanking regions (upstream and downstream; up to ∼1 kb) of the gene were amplified by PCR and cloned into pKC1139 digested with BamHI/HindIII using Gibson assembly. Sequence-verified clones were introduced into nonmethylating E. coli ET12567/pUZ8002 and transmitted into S. coelicolor via conjugation. Single-crossover strains were selected with 50 μg/mL apramycin, and double crossover was induced through liquid culture at 38°C. Single-colony isolation was conducted through serial dilution spreading on an NA (nutrient agar) plate. Deletion mutants were selected by sensitivity to 50 μg/mL apramycin and confirmed by PCR.

For overexpression of the target genes, the ermE* promoter from pMF23 was fused to each target gene cloned into pSET152 digested with BamHI/EcoRV using Gibson assembly. Sequence-verified clones were introduced into S. coelicolor via conjugation with E. coli ET12567/pUZ8002 and selected by resistance to 50 μg/mL apramycin. Plasmids and primers used in this study are listed in Table S1A and B.

### Disk diffusion assay.

For the disk diffusion assay, 1 × 10^8^ spores of wild-type S. coelicolor were overlaid on fresh NA plates. After air drying, 8-mm paper disks (Advantec) were put on the plate. Then, 20 μL of kanamycin (25 μg) and/or FeCl_3_ (15 μg) was dropped on the disk. After incubation at 30°C for 48 h, an inhibition zone was observed.

### Antibiotic sensitivity assay.

To enumerate antibiotic sensitivity, relative growth (%) and number of CFU (CFU/mL) were determined. Antibiotic and iron treatments were done at various concentrations using the early exponential-phase cells. After 8 h (for S. coelicolor) or 16 h (for M. smegmatis) of treatment, relative growth (%) was calculated by determining the ratio of the OD_600_ value of the culture to that of the nontreated control. Along with OD_600_ measurements, aliquots from cultures were subjected to viable counts on NA plates. CFU/mL were enumerated after 48 h of incubation.

### Intracellular iron quantification.

Intracellular iron quantification was conducted as described previously (48) with slight modification. Briefly, the cells were lysed by ice-cold phosphate buffer saline using sonication. Cell lysate (100 μL) was mixed with 100 μL of 10 mM HCl and an iron-releasing solution (2.25% [wt/vol] KMnO_4_, 0.7 M HCl), incubated at 60°C for 2 h, and then subjected to cooling to room temperature, followed by the addition of 30 μL of an iron-releasing solution (6.5 mM ferrozine, 6.5 mM neocuproine, 2.5 M ammonium acetate, 1 M ascorbic acid). After 30 min of incubation, OD_550_ values were measured and converted to intracellular iron levels based on the standard curve.

### Determining MIC and MLC.

An MIC assay was conducted as described previously (49) with slight modification. Early exponential-phase cells were diluted 1:10 with fresh medium, 200 μL of which was dispensed into a 96-well plate with 2-fold serially diluted antibiotics, with or without 250 μM FeCl_3_. After 12 h of incubation (for S. coelicolor) or 36 h of incubation (for M. smegmatis), 30 μL of 0.03% resazurin solution was added to each well. After an additional 3 h of incubation at 30°C for color change, the lowest concentrations with no color change (i.e., blue color remained unchanged) were scored as the MIC values. The minimum lethal concentration (MLC) was determined by directly plating the content (10 μL) of the wells at concentrations higher than the MIC value. After 48 h of incubation, the lowest concentrations with no colony formation were determined as the MLC values.

### Translation rate measurement.

S. coelicolor cells at the early exponential phase (OD_600_ of ∼0.3) were treated with kanamycin and iron for 30 min, followed by treatment with EasyTag l-[^35^S]methionine (1 μCi) (PerkinElmer) at 30°C for 10 min. The incorporation reaction was quenched by adding methionine (0.5 mg) and the same volume of cold trichloroacetic acid (TCA) solution (30%) with vortexing every 10 min for 30 min. Cells were harvested by centrifugation at 10,000 × *g* at 4°C for 5 min and washed twice with ice-cold acetone. After air drying of residual acetone, resuspended pellets were dot blotted on Whatman filter paper. Phosphor signal was imaged using BAS-2500 (Fujifilm). The radioactivity of the blots was quantified from radiographs using MultiGauge v3.0 (Fujifilm).

### RNA-seq and analysis.

S. coelicolor cells at the early exponential phase (OD_600_ of ∼0.3) were treated with kanamycin and/or iron for 30 min and harvested. The harvested cell pellets were washed with cold phosphate-buffered saline (PBS) and subjected to RNA extraction using SDS and a hot-phenol protocol (46), followed by DNase I treatment (Invitrogen). rRNA was depleted using a Ribo-Zero rRNA removal kit (Epicentre, USA), and the libraries for Illumina sequencing were made with the TruSeq stranded total RNA (NEB Microbe, USA) in accordance with the manufacturer’s protocol. RNA sequencing (RNA-seq) was performed using the Illumina NovaSeq platform by paired-end 100-bp sequencing.

Raw sequencing data were analyzed using the Galaxy platform (https://usegalaxy.org) (50). Briefly, after a quality check using FastQC v0.11.4 and adapter sequence trimming using Trimmomatic v0.38, the reads were mapped to the S. coelicolor A3(2) genome (NC_003888.3) using HISAT2 v2.1.0. Total mapped reads were separated by strand specificity using bamSplit v2.4.0 and sam_merge2 v1.2.0. Strand-specific mapping data were counted using featurecounts v1.6.4. Normalized expression data were analyzed using the DESeq2 package in R for identification of differential expressed genes (DEGs). Functional enrichment of DEGs was conducted using Gene Ontology Resource (http://geneontology.org) (51) and Panther (52) using locus tag and UniProt entry. A heat map and dot plot were illustrated using gplots and ggplot2 package in R.

### NAD/NADH quantification.

For quantification of the oxidized form (NAD^+^) and the reduced form (NADH), a PicoSens NAD/NADH assay kit (Biomax) was used according to the manufacturer’s recommendation. Harvested cells were disrupted by extraction buffer using sonication, and cell debris was centrifuged at 20,000 × *g* at 4°C for 10 min. Proteins in the cell lysate were depleted by using a 10-kDa centrifugal filter (Merck). One half of the cell lysate and the other half that had been incubated at 60°C for 30 min to deplete NAD^+^ were used to determine the quantity of the total (i.e., both NAD^+^ and NADH) NAD and that of NADH, respectively. Each sample was treated with cycling enzymes for 5 min and mixed with a colorimetric probe. After incubation for 1.5 h, the OD_450_ was measured for the NAD/NADH quantity normalized by dry cell weight to calculate the ratio of NAD^+^ and NADH.

### ATP quantification.

The ATP level was quantified using the BacTiter-Glo microbial cell viability assay (Promega) according to the manufacturer’s protocol. After treatment with kanamycin and/or iron for 30 min, 100 μL of culture medium was mixed with an equal volume of BacTiter-Glo reagent on a 96-well plate. After reaction for 5 min at room temperature, the luminescence of each well was recorded to determine the level of ATP based on the standard curve.

### Data availability.

RNA-seq data have been deposited in the Gene Expression Omnibus (GEO) under accession number GSE168567.
