# Supplementary material for: Iron-Induced Respiration Promotes Antibiotic Resistance in Actinomycete Bacteria
Source: mBio. 2022 Mar 31;13(2):e00425-22. doi: 10.1128/mbio.00425-22 (PMC9040825; doi:10.1128/mbio.00425-22)
Supplement: FIG S1 [file mbio.00425-22-sf001.pdf]

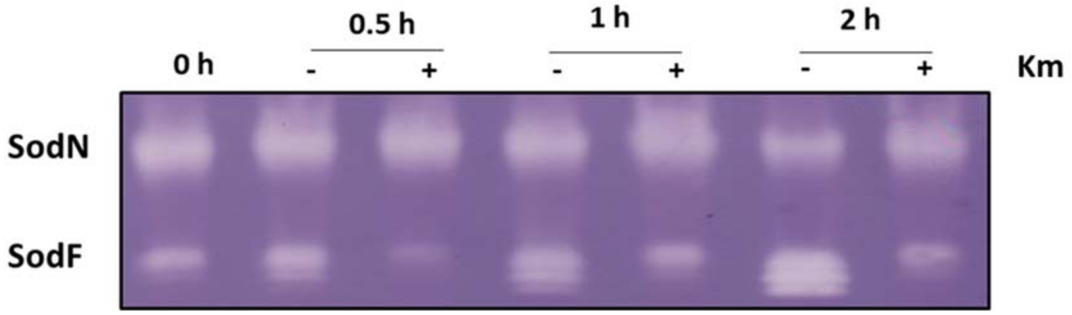

Figure S1. Kanamycin treatment and superoxide dismutase activity

Native-PAGE was performed for activity staining using twenty micrograms of cell lysates from *S. coelicolor* that had been treated for the indicated time (0.5, 1, and 2 h) with (+) or without (-) 1 μg/ml kanamycin (Km). The activity bands of Ni-containing (SodN) and Fe-containing (SodF) SODs were indicated at the left.
