# Supplementary material for: Iron-Induced Respiration Promotes Antibiotic Resistance in Actinomycete Bacteria
Source: mBio. 2022 Mar 31;13(2):e00425-22. doi: 10.1128/mbio.00425-22 (PMC9040825; doi:10.1128/mbio.00425-22)
Supplement: FIG S2 [file mbio.00425-22-sf002.pdf]

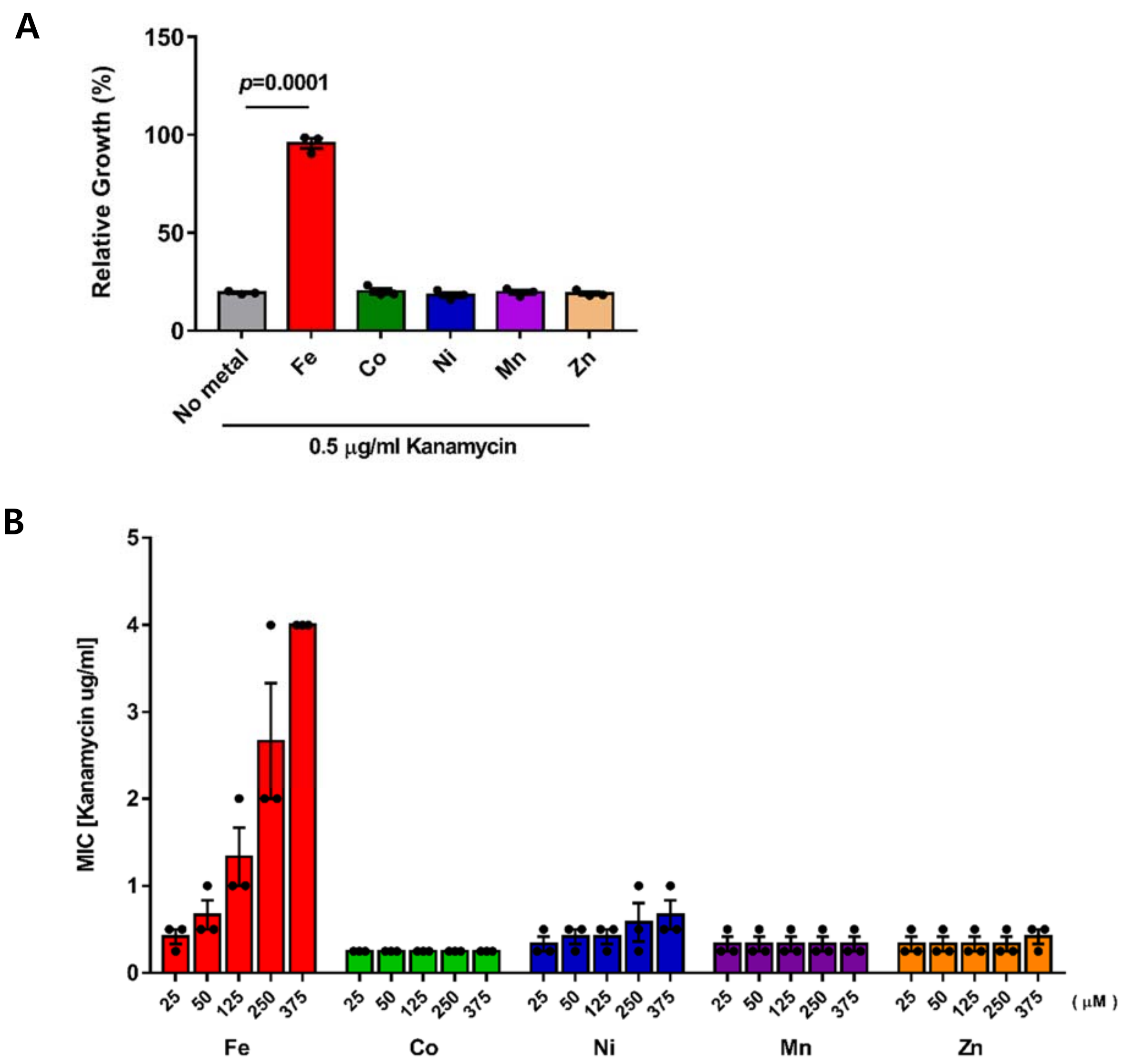

Figure S2. Specificity of iron in promoting kanamycin resistance.

(A) Relative growth (%) was calculated using the OD_{600} values after 8 h of treatment with 0.5 µg/ml kanamycin in the absence or presence of various metals (Fe; $FeCl_3$, Co; $CoCl_2$, Ni; $NiSO_4$, Mn; $MnCl_2$, Zn; $ZnCl_2$) at 250 µM.

(B) MIC values of kanamycin in the presence of various metals at the designated concentrations.

The values shown are the means with the error bars representing the standard deviations from three independent experiments. *p* value was indicated from 2-way ANOVA with Dunnett's multiple comparison test.
