# Supplementary material for: Iron-Induced Respiration Promotes Antibiotic Resistance in Actinomycete Bacteria
Source: mBio. 2022 Mar 31;13(2):e00425-22. doi: 10.1128/mbio.00425-22 (PMC9040825; doi:10.1128/mbio.00425-22)
Supplement: FIG S3 [file mbio.00425-22-sf003.pdf]

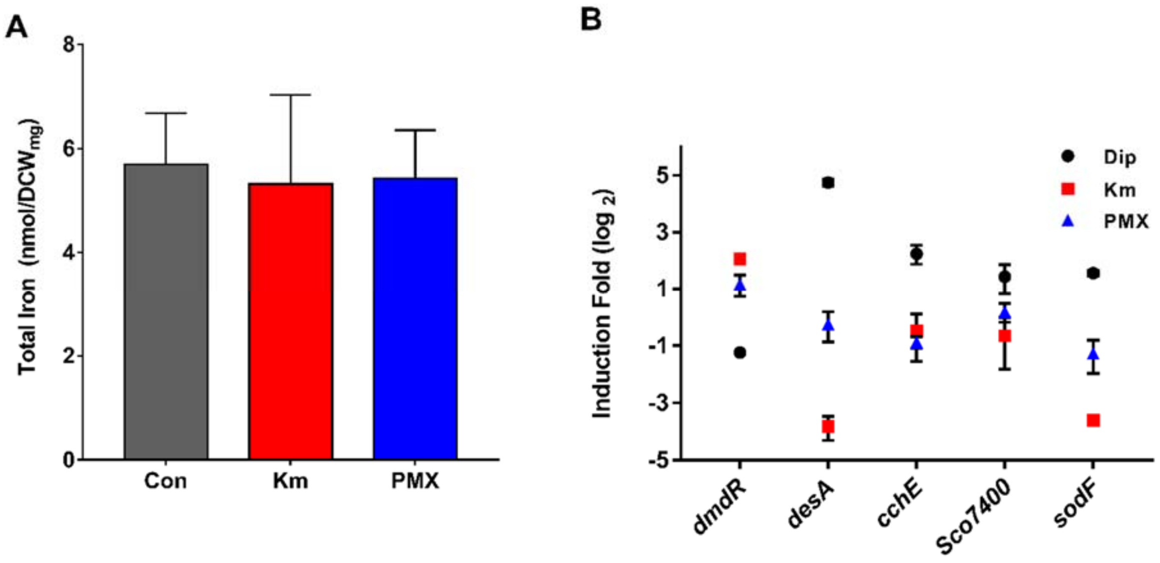

Figure S3. Antibiotic treatment and intracellular iron status

(A) Intracellular total iron contents were measured before (control) and after treatments with 0.5 µg/ml kanamycin (Km; red), or 8 µg/ml polymyxin B (PMX; blue) for 30 min. Measured values were normalized by dried cell weight (DCW_{mg}).

(B) Expression levels of genes for iron regulator (*dmdR*), dmdR regulon (*desA, cchE, sco7400*), or iron-containing enzyme (*sodF*) were measured after treatment with 100 µM dipyridyl (Dip; black), 0.5 µg/ml kanamycin (Km; red), or8 µg/ml polymyxin B (PMX; blue), for 30 min. Gene expression was quantified by using qRT-PCR, and induction folds were calculated relative to non-treated control.

The values are the means with the error bars representing the standard deviations from three independent experiments.
