# Supplementary material for: Iron-Induced Respiration Promotes Antibiotic Resistance in Actinomycete Bacteria
Source: mBio. 2022 Mar 31;13(2):e00425-22. doi: 10.1128/mbio.00425-22 (PMC9040825; doi:10.1128/mbio.00425-22)
Supplement: FIG S4 [file mbio.00425-22-sf004.pdf]

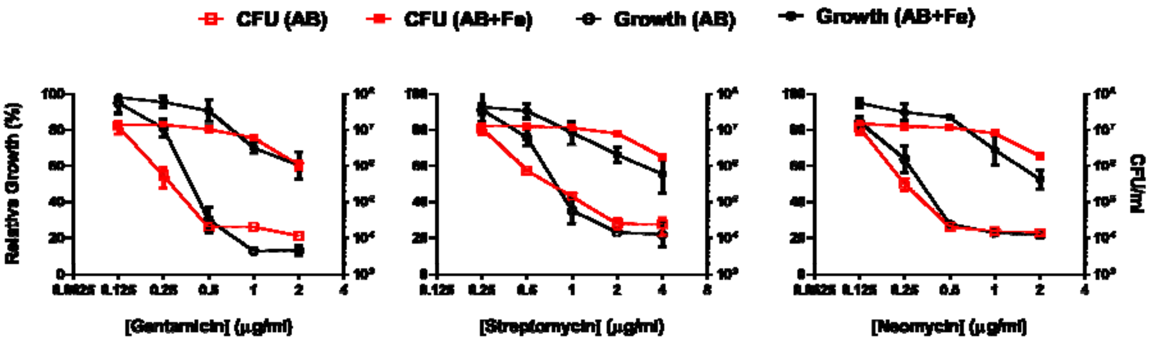

Figure S4. Iron promotes resistance to aminoglycoside drugs

Relative growth (black circle; left axis) and colony forming units (CFUs, red square; right axis) of *S. coelicolor* cells cultured in the presence of gentamicin, streptomycin, or neomycin at designated concentrations with (AB+Fe, filled symbol) or without (AB, open symbol) iron were shown.
