# Supplementary material for: Iron-Induced Respiration Promotes Antibiotic Resistance in Actinomycete Bacteria
Source: mBio. 2022 Mar 31;13(2):e00425-22. doi: 10.1128/mbio.00425-22 (PMC9040825; doi:10.1128/mbio.00425-22)
Supplement: FIG S5 [file mbio.00425-22-sf005.pdf]

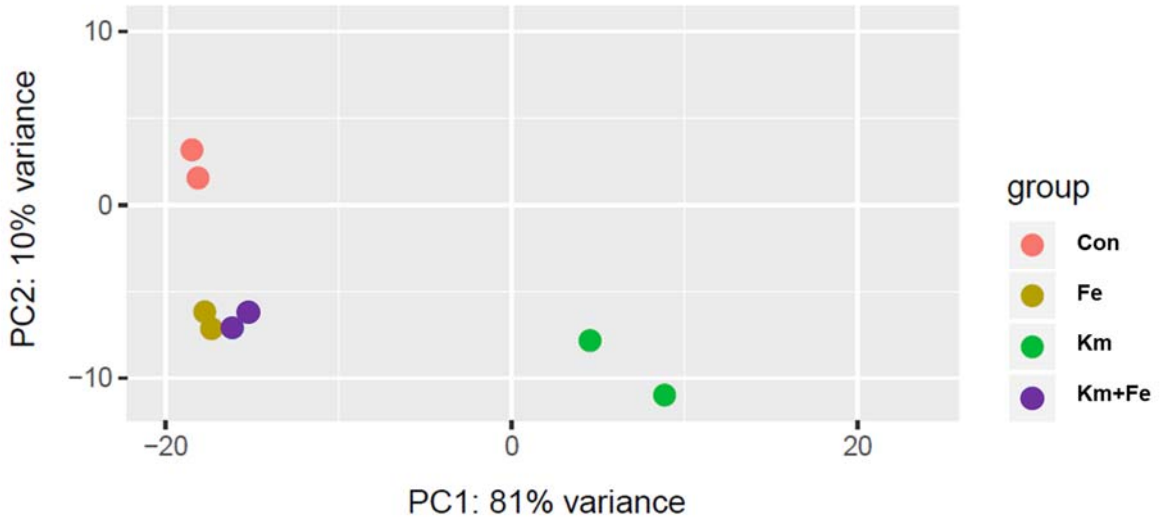

Figure S5. Principal component analysis (PCA) on differential expression datasets from *S. coelicolor*

PCA plot of RNA-seq datasets for the gene expression changes in *S. coelicolor* cells that were untreated (red; control) or treated with 250 μM $FeCl_3$ (yellow), 0.5 μg/ml kanamycin (green), or 0.5 μg/ml kanamycin + 250 μM $FeCl_3$ (purple). Each dataset consisted of two independent transcriptome data (spot). The PCA was performed by using normalized RNA-seq data of 8,152 genes differentially expressed in at least one pairwise comparison: control vs. iron and/or kanamycin treatment.
