# Supplementary material for: Iron-Induced Respiration Promotes Antibiotic Resistance in Actinomycete Bacteria
Source: mBio. 2022 Mar 31;13(2):e00425-22. doi: 10.1128/mbio.00425-22 (PMC9040825; doi:10.1128/mbio.00425-22)
Supplement: FIG S6 [file mbio.00425-22-sf006.pdf]

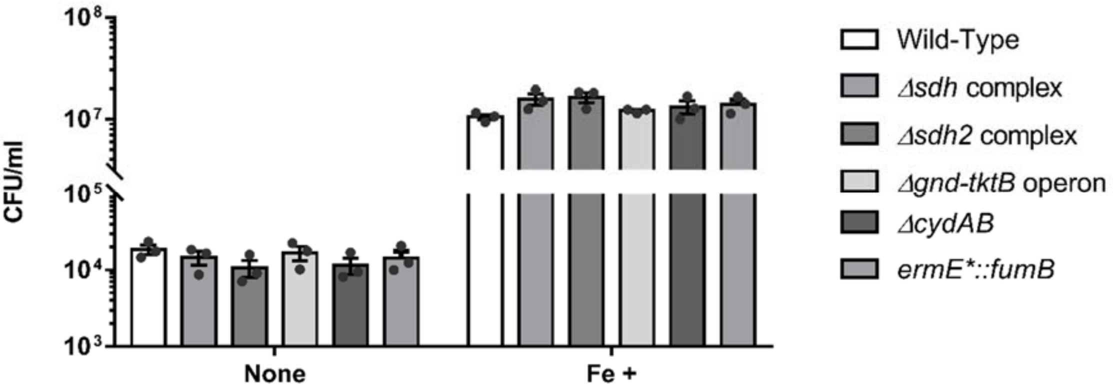

Figure S6. Effect of deletion or overexpression of some metabolic genes on iron-promoted kanamycin resistance.

CFU/ml was enumerated by counting viable cells of wild type, deletion mutant, or overexpression strains after 8-h culture in the presence of kanamycin (0.5 μg/ml) treatment with (Fe+) or without (none) $FeCl_3$ (250 μM) supplementation. The examined strains are mutants deleted of SCO4855-4858 (*Δsdh* complex), SCO0922-0924 (*Δsdh2* complex), SCO6658-6663 (*Δgnd-tktB* operon), and SCO3945-3946 (*ΔcydAB*), and an overexpression strain of SCO5044 driven by ermE* promoter (*ermE*::fumB*). The values are the means with error bars representing the standard deviations from three independent experiments.
