# Supplementary material for: Iron-Induced Respiration Promotes Antibiotic Resistance in Actinomycete Bacteria
Source: mBio. 2022 Mar 31;13(2):e00425-22. doi: 10.1128/mbio.00425-22 (PMC9040825; doi:10.1128/mbio.00425-22)
Supplement: TABLE S2 [file mbio.00425-22-st002.pdf]

Table S2. List of 144 genes responsive to iron

Locus tag	Uniprot entry	Gene Name	Protein name	Fe/Con		Km+Fe/Km		Km/Con		Km+Fe/Con	
				Fold change (log_2)	p adj value	Fold change (log_2)	p adj value	Fold change (log_2)	p adj value	Fold change (log_2)	p adj value
SCO0379	Q9RJK9	*catA*	Catalase	0.770	2.98E-05	1.520	1.92E-13	-0.728	8.70E-06	0.750	6.89E-04
SCO0459	Q9RL28	-	FAD-binding FR-type domain-containing protein	-3.586	1.22E-29	-1.622	3.75E-03	-1.892	1.92E-10	-3.562	3.54E-26
SCO0493	Q9RK13	-	Putative ABC-transporter transmembrane protein	-2.742	1.29E-32	-1.404	6.43E-04	-1.694	1.56E-11	-3.138	1.56E-33
SCO0572	Q93RZ8	-	Putative cytosine permease	0.842	3.18E-03	1.042	8.80E-04	-0.246	6.06E-01	0.755	3.27E-02
SCO0607	Q9RJN7	-	Hypothetical lipoportein	1.510	3.17E-06	1.105	3.12E-03	0.462	4.09E-01	1.525	8.49E-06
SCO0922	Q9RCY8	*sdhB2*	Putative reductase iron-sulfur protein	1.437	3.74E-12	3.127	3.50E-41	-1.543	1.87E-10	1.542	5.87E-13
SCO0923	Q9RCY7	*sdhA2*	Putative reductase flavoprotein subunit	1.606	6.07E-22	3.457	2.30E-37	-1.734	5.55E-04	1.684	1.01E-21
SCO0924	Q9RCY6	*sdhC2*	Putative cytochrome B subunit	1.572	2.78E-16	3.418	7.45E-42	-1.643	1.26E-11	1.734	9.82E-20
SCO1235	Q9FCD2	*ureB*	Urease subunit beta	0.589	3.85E-02	1.803	3.44E-11	-1.112	7.15E-06	0.649	3.36E-02
SCO1245	Q9FCC2	*bioA*	Adenosylmethionine-8-amino-7-oxononanoate aminotransferase	-0.725	1.49E-02	-0.796	6.03E-03	0.129	7.46E-01	-0.708	3.14E-02
SCO1246	Q9FCC1	*bioD*	ATP-dependent dethiobiotin synthetase BioD	-1.036	4.32E-02	-0.937	3.89E-02	-0.016	9.88E-01	-0.994	6.88E-02
SCO1261	Q9K3H9	-	Putative transcriptional regulatory protein	-1.098	1.85E-02	-0.961	2.52E-02	0.127	8.50E-01	-0.876	1.46E-01
SCO1480	Q9KXR9	-	Uncharacterized protein	0.569	1.34E-02	0.864	4.66E-04	-0.051	9.01E-01	0.771	3.34E-03
SCO1567	Q9L1B4	*pqrB*	PqrB (Putative transmembrane-transport protein)	1.181	2.89E-05	-1.219	4.46E-04	2.363	2.00E-04	1.102	4.26E-04

SCO1568	Q9L1B3	-	Putative TetR-family transcriptional regulator	1.245	3.46E-04	-1.314	9.34E-04	2.575	3.63E-15	1.220	5.94E-03
SCO1623	Q9RJ78	-	Uncharacterized protein	-1.487	1.10E-11	-0.681	4.51E-02	-0.671	6.90E-03	-1.394	4.35E-08
SCO1648	Q9RJ58	*arc*	Proteasome-associated ATPase	0.923	8.40E-06	0.730	2.96E-03	-0.095	7.88E-01	0.594	2.20E-02
SCO1700	Q9S252	-	Putative membrane protein	2.196	4.51E-08	1.465	1.42E-03	1.063	1.03E-01	2.482	3.35E-09
SCO1780	Q9S220	*recN*	DNA repair protein RecN	-0.861	4.91E-05	-0.843	3.12E-03	0.011	9.84E-01	-0.873	3.34E-03
SCO1785	Q9S215	-	Putative iron-siderophore uptake system ATP-binding component	-3.322	7.73E-37	-8.174	3.87E-03	5.224	9.62E-02	-2.973	5.14E-26
SCO1786	Q9S214	-	Putative iron-siderophore uptake system transmembrane component	-3.065	1.46E-37	-1.448	1.64E-04	-1.494	1.95E-11	-2.980	1.59E-26
SCO1787	Q9S213	-	Putative iron-siderophore uptake system transmembrane component	-2.365	9.36E-36	-1.245	8.65E-06	-1.138	5.72E-10	-2.424	1.42E-28
SCO1865	Q93RW1	*ectB*	Diaminobutyrate--2-oxoglutarate transaminase	0.711	1.88E-04	0.630	1.37E-02	-0.094	7.81E-01	0.495	1.34E-01
SCO1866	Q93RW0	*ectC*	L-ectoine synthase	0.614	9.48E-03	0.688	6.05E-03	-0.315	2.02E-01	0.333	5.24E-01
SCO1867	Q93RV9	*ectD*	Ectoine dioxygenase	0.627	1.03E-02	0.861	7.71E-04	-0.424	8.17E-02	0.395	4.30E-01
SCO1875	Q93RV1	-	Putative secreted penicillin binding protein	0.603	1.81E-02	-1.220	1.98E-07	2.108	2.75E-29	0.846	6.18E-04
SCO1876	Q93RV0	-	Putative RNA polymerase sigma factor	1.041	4.78E-02	-2.226	2.67E-13	2.972	8.37E-25	0.710	5.14E-01
SCO1878	Q93RU8	-	Putative secreted protein	0.715	1.65E-02	1.508	3.24E-07	-0.668	4.56E-02	0.799	1.38E-02
SCO1904	Q9X9W8	-	Putative transcriptional regulator	-1.137	4.06E-02	-1.840	4.77E-08	1.521	4.17E-07	-0.360	8.60E-01
SCO1905	Q9X9W7	-	Uncharacterized protein	2.065	9.40E-05	-3.005	6.64E-31	6.520	5.95E-99	3.472	6.86E-20
SCO1963	Q9Z503	-	Putative integral membrane export protein	-0.920	1.79E-04	-1.209	6.09E-05	0.382	1.24E-01	-0.869	1.39E-02
SCO2000	Q9S2K3	-	Putative ATP-binding RNA helicase	-0.956	9.62E-03	-1.316	7.05E-04	0.315	4.19E-01	-1.042	3.83E-02

SCO2025	Q9S2Z0	-	Putative glutamate synthase small subunit	0.941	1.50E-07	0.754	1.03E-03	0.003	9.96E-01	0.716	1.23E-03
SCO2026	Q9S2Y9	-	Putative glutamate synthase large subunit	1.220	6.41E-16	0.908	1.34E-05	0.214	3.19E-01	1.080	3.71E-09
SCO2027	Q9S2Y8	-	Putative membrane protein	3.362	8.92E-25	1.649	1.60E-06	1.527	2.69E-03	3.132	1.36E-18
SCO2101	Q9S2V5	-	Putative carotenoid dehydrogenase	-1.261	3.80E-04	-1.000	7.07E-03	0.012	9.90E-01	-1.029	9.96E-03
SCO2113	Q9S2N0	*bfr*	Bacterioferritin (BFR)	2.733	3.33E-22	1.498	1.94E-07	1.616	6.29E-06	3.073	1.59E-26
SCO2114	Q9S2M9	-	Fer2_BFD domain-containing protein	-1.861	1.17E-11	-1.335	1.78E-03	-0.751	1.27E-02	-2.124	1.14E-10
SCO2171	Q9S2R5	-	Putative secreted protease	-0.853	4.01E-02	-1.976	4.76E-12	0.914	2.21E-01	-1.105	4.02E-03
SCO2299	Q9L014	-	Putative bifunctional protein (Ribonuclease H/phosphoglycerate mutase)	-0.588	3.14E-02	-0.571	3.86E-02	-0.052	9.11E-01	-0.664	2.87E-02
SCO2310	Q8CJZ9	-	Putative integral membrane efflux protein	0.622	1.14E-02	-0.700	2.26E-02	1.370	2.43E-09	0.628	2.15E-02
SCO2448	Q9L098	-	Uncharacterized protein	-0.724	5.27E-03	-0.651	3.99E-02	0.029	9.55E-01	-0.663	7.41E-02
SCO2462	Q8CJZ5	-	Putative sugar kinase	-0.783	1.08E-03	-0.667	1.21E-02	-0.006	9.92E-01	-0.714	9.39E-03
SCO2505	Q9L2H8	*znuA*	High-affinity zinc uptake system protein ZnuA	1.629	7.52E-17	2.126	5.24E-18	-0.611	3.34E-02	1.474	6.19E-12
SCO2552	Q9RDD9	-	Ribosomal RNA small subunit methyltransferase E	-0.896	1.08E-02	-0.990	1.89E-03	0.280	4.66E-01	-0.753	9.48E-02
SCO2672	Q9L249	-	Putative membrane protein	-0.817	4.13E-02	-0.961	4.79E-03	0.400	2.19E-01	-0.602	3.29E-01
SCO2780	Q9L074	*desE*	Putative secreted protein	-3.168	5.78E-56	-1.415	5.88E-06	-1.989	1.91E-23	-3.445	1.58E-57
SCO2781	Q9L073	*desF*	FAD-binding FR-type domain-containing protein	-4.274	2.97E-29	-1.927	9.70E-04	-1.816	1.49E-08	-3.788	4.15E-23
SCO2782	Q9L072	*desA*	Putative pyridoxal-dependent decarboxylase	-4.445	1.02E-29	-1.847	1.94E-02	-2.588	5.73E-15	-4.480	3.49E-25

SCO2785	Q9L069	*desD*	Uncharacterized protein	-3.895	3.61E-27	-1.847	1.71E-03	-1.858	8.13E-09	-3.742	2.10E-23
SCO2806	Q9F3H5	-	Putative mutase	-0.979	5.61E-03	-0.845	2.06E-02	0.097	8.60E-01	-0.790	8.53E-02
SCO2893	Q9KZR0	-	Uncharacterized protein	-0.923	1.71E-02	-0.987	3.95E-03	0.501	1.33E-01	-0.527	4.89E-01
SCO2985	Q9L050	-	Putative integral membrane protein	-0.885	6.56E-03	-0.881	1.13E-02	-0.066	9.08E-01	-0.989	4.02E-03
SCO3037	Q9KZK8	*fbiB*	Bifunctional F420 biosynthesis protein FbiB	-0.621	2.55E-02	-0.574	4.40E-02	-0.058	8.88E-01	-0.673	2.21E-02
SCO3043	Q9KZK2	-	Uncharacterized protein	0.503	3.67E-02	1.149	1.27E-05	-0.593	7.92E-03	0.514	1.69E-01
SCO3048	Q93J54	-	Putative membrane protein	1.491	1.24E-15	1.510	4.15E-11	-0.117	7.81E-01	1.352	2.13E-10
SCO3050	Q93J52	-	Uncharacterized protein	0.673	3.24E-02	0.704	3.05E-02	0.134	7.91E-01	0.796	2.30E-02
SCO3079	Q9KZ69	-	Putative thiolase	0.670	3.59E-02	0.693	1.99E-02	-0.041	9.45E-01	0.612	1.48E-01
SCO3086	Q9KZ62	-	Putative lipoprotein	2.868	2.87E-36	2.789	4.41E-28	0.337	5.47E-01	3.083	2.00E-37
SCO3090	Q9KZ59	-	Putative ABC transporter integral membrane protein	1.219	5.64E-09	0.637	1.23E-02	0.585	6.90E-03	1.181	4.99E-08
SCO3110	Q9F2P0	-	Putative ABC transport system integral membrane protein	1.038	3.55E-08	0.776	1.62E-03	0.280	2.92E-01	1.013	6.77E-06
SCO3127	Q9RNU9	*ppc*	Phosphoenolpyruvate carboxylase (PEPC)	0.693	2.77E-04	1.292	1.45E-07	-0.683	3.53E-04	0.569	9.44E-02
SCO3166	Q9RKC1	-	Putative membrane transport protein	1.121	7.58E-08	-1.216	1.42E-05	2.079	1.10E-19	0.820	2.64E-03
SCO3194	Q9KYV1	-	Putative lipoprotein	1.277	3.07E-13	1.450	2.19E-11	-0.217	3.97E-01	1.192	4.28E-09
SCO3195	Q9KYV0	-	Uncharacterized protein	0.879	2.46E-02	-0.847	1.48E-02	1.735	7.20E-10	0.847	7.84E-02
SCO3286	Q9X878	-	Putative secreted protein	3.124	1.00E-41	-1.093	2.39E-03	4.182	8.05E-13	3.044	4.21E-31
SCO3299	Q9X891	-	3'-phosphate/5'-hydroxy nucleic acid ligase	0.871	7.32E-03	-3.600	9.21E-57	5.148	1.02E-165	1.505	1.37E-07
SCO3317	Q9WX17	-	Putative uroporphyrin-III C-methyltransferase/uroporphyrinogen-III synthase	-0.595	5.97E-03	0.665	7.43E-03	-1.175	3.74E-11	-0.551	4.21E-02

SCO3542	Q9X908	*tmk*	Thymidylate kinase	-0.607	3.11E-03	-0.685	6.88E-03	-0.009	9.85E-01	-0.735	5.05E-03
SCO3590	Q8CJW1	-	Putative two-component system response regulator	2.203	1.38E-15	1.117	5.92E-04	0.630	1.33E-01	1.706	2.70E-08
SCO3592	Q9XAL0	-	Putative membrane protein	3.189	8.63E-23	2.712	1.90E-10	-0.050	9.77E-01	2.615	1.42E-11
SCO3593	Q9XAK9	-	Uncharacterized protein	3.340	1.91E-18	2.945	2.54E-11	-0.152	9.31E-01	2.754	1.23E-09
SCO3594	Q9XAK8	-	Putative D-lactate dehydrogenase	4.465	8.56E-23	4.294	2.92E-10	-0.776	NA	3.461	4.58E-10
SCO3607	Q9XAJ5	-	Putative secreted protein	4.140	5.78E-56	0.914	2.05E-02	3.080	5.32E-16	3.950	3.54E-46
SCO3712	Q9X8Z4	-	Putative hydrolase	0.982	2.67E-04	0.949	1.54E-03	0.110	8.50E-01	1.018	6.95E-04
SCO3899	Q9X8T5	-	Uncharacterized protein	0.610	1.71E-02	1.512	9.21E-11	-0.864	1.51E-05	0.607	2.88E-02
SCO3900	Q9X8T6	-	Uncharacterized protein	0.602	3.17E-03	1.367	8.35E-10	-0.757	3.79E-05	0.569	3.35E-02
SCO3945	Q9ZBY7	*cydA*	Putative cytochrome oxidase subunit I	0.685	3.40E-03	2.526	2.16E-23	-1.684	8.86E-14	0.801	4.17E-04
SCO3946	Q9ZBY6	*cydB*	Putative cytochrome oxidase subunit II	0.812	1.90E-04	2.320	1.66E-19	-1.388	1.12E-08	0.890	4.41E-04
SCO3968	Q93J38	-	Putative integral membrane protein	-0.855	8.68E-04	-0.818	2.35E-03	-0.575	1.62E-02	-1.434	3.03E-09
SCO3977	Q93J30	-	Putative protease (Putative secreted protein)	0.745	8.96E-05	-0.959	4.00E-05	1.799	1.93E-30	0.800	1.60E-03
SCO4005	Q9ADQ0	-	Putative RNA polymerase sigma factor	3.613	8.94E-37	1.818	1.31E-10	1.743	2.95E-05	3.517	1.91E-29
SCO4006	Q9ADP9	-	Putative fatty acid CoA ligase	2.909	7.67E-36	1.730	1.73E-10	0.837	2.49E-02	2.524	3.68E-23
SCO4020	Q9ADN7	-	Putative two component system response regulator	1.133	1.19E-06	-2.218	4.18E-26	3.327	3.53E-79	1.069	1.07E-04
SCO4021	Q9ADN6	-	Histidine kinase	1.044	1.66E-05	-2.333	4.36E-25	3.416	3.13E-88	1.042	5.89E-04
SCO4022	Q9ADN5	-	Putative glycosyl transferase	1.914	3.04E-04	-1.223	7.69E-04	3.041	3.36E-16	1.775	2.24E-03
SCO4030	Q9ADM8	-	Uncharacterized protein	1.609	1.13E-04	1.184	1.41E-02	0.560	4.61E-01	1.700	7.97E-05
SCO4031	Q9ADM7	-	Putative integral membrane transport protein	2.242	1.98E-36	1.169	7.29E-05	1.069	6.73E-05	2.196	1.39E-26
SCO4032	Q9ADM6	-	Putative marR regulatory protein	2.362	2.89E-21	1.381	1.05E-05	0.960	1.26E-02	2.298	1.11E-18

SCO4049	Q9AK68	-	Putative antibiotic binding protein	-2.886	5.23E-23	-1.382	8.81E-03	-1.765	7.19E-10	-3.198	2.51E-22
SCO4156	Q9KZU5	-	Putative two-component systen response regulator	1.342	2.06E-08	-1.924	3.98E-18	3.219	6.54E-67	1.253	1.56E-06
SCO4157	Q9KZU4	-	Putative protease	1.490	4.00E-11	-1.790	5.51E-17	3.403	1.61E-80	1.571	4.70E-11
SCO4159	Q05943	*glnR*	Transcriptional regulatory protein GlnR	0.589	1.59E-02	0.646	1.21E-02	-0.106	7.81E-01	0.498	1.63E-01
SCO4163	Q9KZT9	-	Putative secreted protein	0.637	1.65E-02	0.795	8.56E-03	-0.055	9.18E-01	0.698	5.21E-02
SCO4198	Q9FCH1	-	Putative DNA-binding protein	0.622	2.60E-02	0.938	9.80E-04	-0.139	7.50E-01	0.760	2.60E-02
SCO4199	Q9FCH0	-	Uncharacterized protein	0.617	9.02E-03	0.902	1.87E-03	-0.111	7.79E-01	0.751	2.09E-02
SCO4289	Q9KXV2	-	Putative secreted protein	1.908	1.99E-07	1.444	2.18E-03	0.109	9.27E-01	1.512	2.05E-03
SCO4330	Q9KXM7	-	Uncharacterized protein	-0.730	1.07E-02	-0.603	4.96E-02	-0.220	4.74E-01	-0.865	2.45E-03
SCO4394	Q7AKJ7	*dmdR1*	DmdR1 protein (Iron repressor)	-0.553	4.17E-02	-0.667	8.30E-03	0.292	2.27E-01	-0.416	3.35E-01
SCO4631	Q9L0M9	-	HNHc domain-containing protein	0.532	4.86E-02	0.912	1.29E-03	-0.344	2.10E-01	0.526	2.27E-01
SCO4896	Q9AK30	-	Putative transport integral membrane protein	0.602	2.08E-02	-0.847	8.69E-03	1.447	2.61E-09	0.558	1.63E-01
SCO4908	Q8R5N3	-	Putative RNA polymerase sigma factor	3.776	5.78E-56	3.149	1.45E-34	0.733	9.98E-02	3.839	2.88E-59
SCO5044	Q9FBN4	*fumB*	Fumarate hydratase class I	0.779	8.78E-06	0.871	4.57E-05	0.014	9.74E-01	0.844	2.74E-05
SCO5147	Q9FBL1	-	Putative ECF-subfamily sigma factor	0.950	9.59E-06	-1.464	4.06E-11	2.479	8.40E-44	0.973	2.12E-05
SCO5178	Q9FCL0	-	Putative sulfurylase	0.657	7.42E-04	0.812	5.04E-04	-0.096	7.64E-01	0.674	7.33E-03
SCO5190	Q9FCJ8	*whiB7/wblC*	Transcriptional regulator WhiB	1.349	2.24E-08	-3.975	6.25E-47	6.086	1.39E-38	2.068	5.48E-21
SCO5191	Q9FCJ7	-	Uncharacterized protein	1.555	2.20E-08	-4.052	1.09E-53	6.311	1.39E-54	2.217	2.31E-21
SCO5205	Q9K4B3	-	Uncharacterized protein	-0.701	4.00E-02	-1.445	8.39E-06	0.599	2.98E-02	-0.886	4.59E-02
SCO5227	Q9K3B9	-	Putative redoxin	-0.696	1.15E-02	-0.826	1.03E-02	-0.013	9.83E-01	-0.879	9.72E-03
SCO5254	P80735	*sodN*	Superoxide dismutase [Ni] (NiSOD)	1.075	2.43E-10	1.949	1.54E-19	-0.701	4.01E-05	1.206	4.28E-09
SCO5365	Q9K4D9	-	Putative transferase	0.564	3.46E-02	0.564	2.79E-02	-0.346	1.11E-01	0.177	8.91E-01
SCO5397	Q9L2C3	-	Large Ala/Glu-rich protein	-1.158	1.70E-10	-0.562	2.61E-02	-0.434	3.10E-02	-1.039	1.77E-06

SCO5402	Q9L2B8	-	Putative Asp-rich hydrophilic protein	1.482	3.06E-03	1.413	1.44E-02	-0.372	7.25E-01	1.001	2.16E-01
SCO5515	Q9Z564	-	D-3-phosphoglycerate dehydrogenase	0.712	1.36E-04	1.258	4.82E-09	-0.613	9.75E-04	0.604	1.56E-02
SCO5676	O86823	-	Putative 4-aminobutyrate aminotransferase	0.681	1.06E-02	0.863	1.27E-04	-0.161	6.12E-01	0.662	1.08E-02
SCO5829	O70005	-	Histidine kinase	-1.419	1.87E-04	-1.453	2.56E-03	-0.483	2.67E-01	-1.980	1.21E-06
SCO5830	O70006	-	Uncharacterized protein	-2.056	2.78E-11	-1.468	1.48E-04	-0.428	2.08E-01	-1.939	7.13E-09
SCO5862	P0A4I1	*cutR*	Transcriptional regulatory protein CutR	0.803	1.27E-03	-1.144	1.53E-06	1.900	1.11E-23	0.714	2.19E-02
SCO5863	P0A4I7	*cutS*	Sensor protein CutS	0.688	2.93E-03	-1.106	5.70E-07	1.805	2.74E-26	0.658	2.15E-02
SCO5881	Q7AKF4	-	Response regulator	0.774	9.02E-03	1.006	1.34E-04	-0.189	6.41E-01	0.775	1.20E-02
SCO5999	Q7AKF3	*sacA*	Aconitate hydratase (Aconitase)	0.526	4.87E-03	0.990	3.72E-06	-0.459	8.19E-03	0.490	6.67E-02
SCO6019	O69849	-	Uncharacterized protein	-0.880	4.24E-05	-0.794	1.53E-03	0.084	8.18E-01	-0.752	2.59E-03
SCO6158	Q9ZBT0	-	Uncharacterized protein	-3.595	3.22E-21	-2.553	7.21E-05	-1.647	3.44E-07	-4.258	1.43E-21
SCO6169	Q9ZBP0	-	Putative regulatory protein	-0.762	1.57E-02	-0.899	5.25E-03	0.164	6.71E-01	-0.775	5.22E-02
SCO6353	O86622	-	Histidine kinase	1.343	7.79E-06	1.475	6.91E-07	-0.094	8.88E-01	1.340	1.77E-06
SCO6354	O86623	-	Putative two-component regulator	1.378	2.93E-07	0.932	1.57E-03	0.406	2.92E-01	1.296	5.41E-06
SCO6355	O86624	-	Putative integral membrane protein	1.185	1.15E-05	0.791	1.34E-02	0.465	2.04E-01	1.214	1.10E-05
SCO6357	O86626	-	Putative membrane protein	1.277	7.40E-07	0.623	2.53E-02	0.812	1.59E-03	1.396	1.64E-07
SCO6378	O86670	-	Putative membrane protein	1.318	9.07E-04	1.262	9.01E-04	0.330	6.48E-01	1.551	5.65E-05
SCO6452	Q9ZBG1	-	Putative transport permease protein	-0.550	1.06E-02	-0.827	8.54E-04	0.078	8.14E-01	-0.789	1.78E-03
SCO6658	O88014	*gnd*	6-phosphogluconate dehydrogenase	0.793	1.09E-04	1.428	5.79E-10	-0.358	1.72E-01	1.029	1.44E-06
SCO6659	O88015	*pgi*	Glucose-6-phosphate isomerase 1	0.831	5.24E-05	1.602	4.14E-15	-0.582	1.47E-03	0.979	7.72E-07
SCO6660	O88016	-	Uncharacterized protein	0.694	3.15E-03	1.172	1.39E-06	-0.377	1.62E-01	0.753	4.77E-03
SCO6661	O88017	*zwf*	Glucose-6-phosphate 1-dehydrogenase	0.554	8.03E-03	1.196	4.63E-08	-0.364	9.42E-02	0.791	2.59E-04
SCO6662	O88018	*tal*	Transaldolase 1	0.531	1.74E-02	0.997	8.07E-06	-0.308	1.81E-01	0.648	9.72E-03
SCO6720	Q9X7M6	-	Putative ABC transporter	2.113	1.77E-04	-3.695	3.06E-31	5.563	1.72E-18	1.825	8.75E-03

SCO6751	Q9X7Q7	-	Putative efflux protein	-1.105	2.75E-03	-1.069	3.46E-03	0.116	8.40E-01	-0.993	2.12E-02
SCO7077	Q9KZ54	-	Putative integral membrane protein	0.907	2.66E-02	1.230	9.64E-04	-0.096	9.09E-01	1.092	6.32E-03
SCO7229	Q9K456	-	Putative membrane protein	1.992	4.19E-02	2.091	1.36E-02	0.088	NA	2.140	2.63E-02
SCO7346	Q9KXZ7	-	Putative membrane transport protein	0.815	3.27E-04	0.984	1.35E-04	0.027	9.62E-01	0.969	5.74E-05
SCO7398	Q9L179	-	Putative membrane transport protein	-3.552	3.75E-29	-2.059	1.29E-06	-1.436	3.28E-07	-3.537	5.07E-27
SCO7399	Q9L178	-	Possible binding-protein-dependent transport lipoprotein	-3.260	8.91E-42	-1.377	3.12E-04	-1.859	1.72E-15	-3.275	1.56E-33
SCO7400	Q9L177	-	Putative ABC-transport protein, ATP-binding component	-2.711	2.36E-24	-1.293	5.81E-03	-1.678	1.04E-10	-3.010	4.02E-24
SCO7507	Q93J90	-	Putative dioxygenase	1.935	9.03E-06	-1.234	7.77E-04	3.246	8.72E-19	1.968	1.44E-05
