# Supplementary material for: Iron-Induced Respiration Promotes Antibiotic Resistance in Actinomycete Bacteria
Source: mBio. 2022 Mar 31;13(2):e00425-22. doi: 10.1128/mbio.00425-22 (PMC9040825; doi:10.1128/mbio.00425-22)
Supplement: FIG S7 [file mbio.00425-22-sf007.pdf]

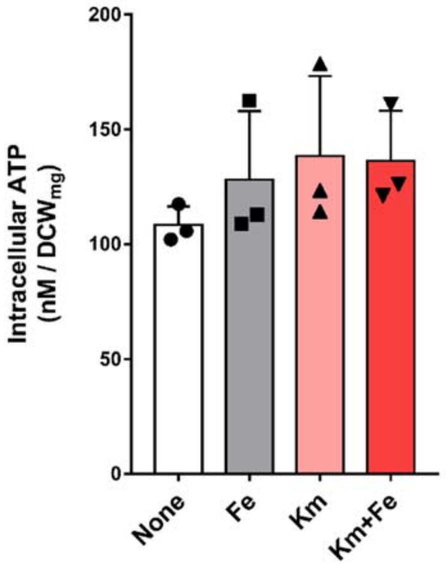

Figure S7. Levels of ATP under kanamycin and iron treatments

Quantification of the intracellular amount of ATP after treatments with 0.5 µg/ml kanamycin (Km) and/or 250 µM iron (Fe) for 30 min. Measurements were normalized by dried cell weight (DCW_{mg}). The values are the means with the error bars representing the standard deviations from three independent experiments.
