# Supplementary material for: Iron-Induced Respiration Promotes Antibiotic Resistance in Actinomycete Bacteria
Source: mBio. 2022 Mar 31;13(2):e00425-22. doi: 10.1128/mbio.00425-22 (PMC9040825; doi:10.1128/mbio.00425-22)
Supplement: FIG S8 [file mbio.00425-22-sf008.pdf]

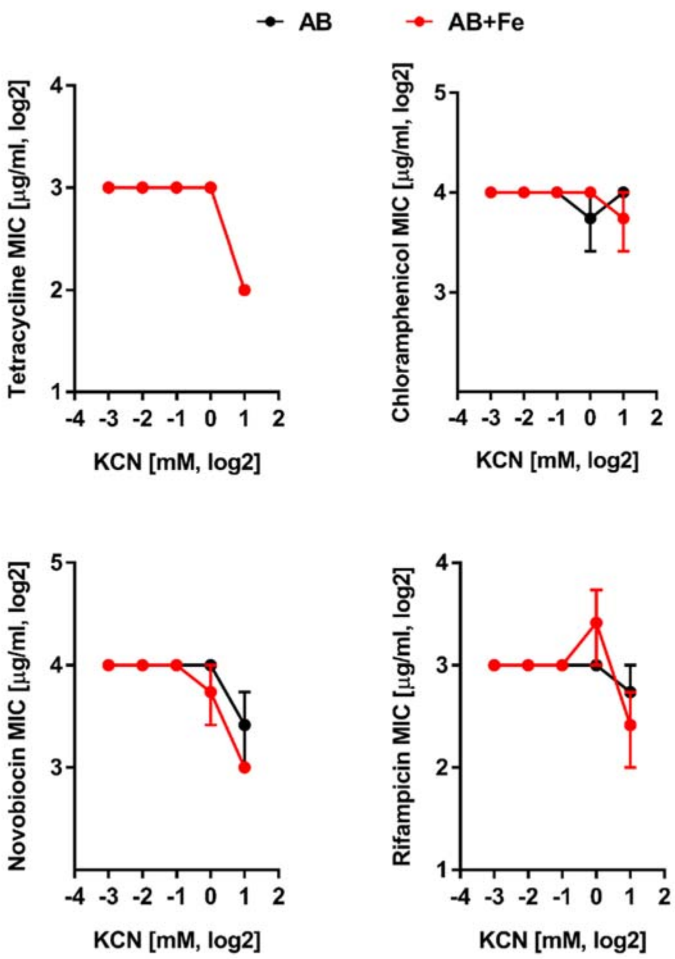


**Figure S8. Effect of KCN on MIC of bacteriostatic antibiotics in the presence or absence of iron supplementation.**

MIC values of bacteriostatic antibiotics by KCN treatment for 30 min. Each MIC values were determined with (AB+Fe) or without iron (AB; black). The values are the means with the error bars representing the standard deviations from three independent experiments.
