# Supplementary material for: Iron-Induced Respiration Promotes Antibiotic Resistance in Actinomycete Bacteria
Source: mBio. 2022 Mar 31;13(2):e00425-22. doi: 10.1128/mbio.00425-22 (PMC9040825; doi:10.1128/mbio.00425-22)
Supplement: TABLE S1 [file mbio.00425-22-st001.pdf]

Table S1. List of strains, plasmids, and primers used in this study

A. Strains and plasmids

Strain or plasmid	Relevant characteristics or purposea	Reference or source
Escherichia coli		
DH5α	For multipurpose cloning	Laboratory stock
ET12567/pUZ8002	For conjugation of plasmid to *Streptomyces*; *dam dcm hsdS*/pUZ8002	Laboratory stock
Streptomyces coelicolor		
M145	*Streptomyces coelicolor* wild-type strain	Laboratory stock
Δ*sdh* complex	*S. coelicolor* Δ*sdh* complex (SCO04855-4858)	This study
Δ*sdh2* complex	*S. coelicolor* Δ*sdh2* complex (SCO0922-0924)	This study
Δ*gnd-tktB* operon	*S. coelicolor* Δ*gnd-tktB* operon (SCO6658-6663)	This study
Δ*cydAB*	*S. coelicolor* Δ*cydAB* (SCO3945-3946)	This study
ermE::fumB*	*S. coelicolor* with pSET*ermE*::fumB*	This study
Others		
M. smegmatis	*Mycobacterium smegmatis* MC2-155 Wild-type	Laboratory stock
C. glutamicum	*Corynebacterium glutamicum* ATCC 13032 Wild-type	Laboratory stock
B. subtilis	*Bacillus subtilis* ATCC 6633 Wild-type	Laboratory stock
V. vulnificus	*Vibrio vulnificus* M06-24/O Wild-type	Laboratory stock
Plasmids		
pSET152	Integrative pMB1 derivative for gene introduction in *Streptomyces*; Am^R	(1)
pKC1139	Containing temperature-sensitive replication origin for gene deletion in *Streptomyces*; Am^R	(2)

pMF23	Containing *ermE*::GUS* for *ermE** promoter-derived overexpression; Am^R	Laboratory stock
pKC-sdh	pKC1139 derivative for deletion of the *sdh* complex (SCO4855-4858); Am^R	This study
pKC-sdh2	pKC1139 derivative for deletion of the *sdh2* complex (SCO0922-0924); Am^R	This study
pKC-gnd tktB	pKC1139 derivative for deletion of the *gnd-tktB* operon (SCO6658-6663); Am^R	This study
pKC-cydAB	pKC1139 derivative for deletion of the *cydAB* gene (SCO3945-3946); Am^R	This study
pSETermE-fumB	pSET152 derivative for *ermE**-driven overexpression of the *fumB* gene (SCO5044); Am^R	This study

[a]: Am^R; Apramycin-resistant

B. Primers

**Primer**[a]	**Sequence**	**Construct / Target gene**
<u>Gene deletion</u>		
SCO sdh UP F	CGTTGTAAAACGACGGCCAGTGCCAGACCTCCAGCCACACCCGCG	pKC-sdh
SCO sdh UP R	GAGGAAATTTCCCACCGTCGGTGTACGCCGGTC	pKC-sdh
SCO sdh DW F	TACACCGACGGTGGGAAATTTCCTCCGGGAGCGG	pKC-sdh
SCO sdh DW R	GCTATGACATGATTACGAATTCGATATAGGACCGGCTCGGGCTGG	pKC-sdh
SCO sdh2 UP F	TGTAAAACGACGGCCAGTGCCACAGCGAGAGGAAGTGCCGCA	pKC-sdh2
SCO sdh2 UP R	ACCGATGAGGCCGCCGTAGGAGTGCTGTTGC	pKC-sdh2
SCO sdh2 DW F	CTCCTACGGCGGCCTCATCGGTCCAAGACATGG	pKC-sdh2
SCO sdh2 DW R	ATGACATGATTACGAATTCGATCCGACATGGCCTCGGCGATG	pKC-sdh2
SCO gnd-tktB UP F	CGTTGTAAAACGACGGCCAGTGCCAGGAACAGCACGTCCACCTGGG	pKC-gnd-tktB
SCO gnd-tktB UP R	CGCTCCGATCGAGGACAACGCCGTGCCGCTGCC	pKC-gnd-tktB
SCO gnd-tktB DW F	CACGGCGTTGTCCTCGATCGGAGCGAAGTAGCTGG	pKC-gnd-tktB
SCO gnd-tktB DW R	GCTATGACATGATTACGAATTCGATTGGCAGTCGTGGCGGACGAT	pKC-gnd-tktB
SCO cydAB UP F	CGTTGTAAAACGACGGCCAGTGCCACTACGCCTGGCGCTCCTTCG	pKC-cydAB
SCO cydAB UP R	TCGGCGAGGTGCGTCTTCACTCTCCTCACATCGCC	pKC-cydAB

SCO cydAB DW F	GGAGAGTGAAGACGCACCTCGCCGACGCCTCGC	pKC-cydAB
SCO cydAB DW R	GCTATGACATGATTACGAATTCGATCGTCCTGGGTAGCGGACCGTC	pKC-cydAB
Gene overexpression		
ermE F	GGGCTGCAGGTCGACTCTAGAGAGCCCGACCCGAGCACGCGC	pSETermE-sacA and pSETermE-fumB
ermE fumB R	ATCTCGCCCATGTCCGTACCTCCGTTGCTCGACTAGACGATCC	pSETermE-fumB
fumB F	GAGGTACGGACATGGGCGAGATGCCTGAGTTCGCGTAC	pSETermE-fumB
fumB R	ATGACATGATTACGAATTCGATTTACGCCAGGCCCGGCCCCC	pSETermE-fumB
qRT-PCR		
RT DmdR F	GGCGACGCGCGTGATGCGCAAG	SCO4394 (dmdR)
RT DmdR R	CCAGGCCCGGGATCGGGTTGCC	
RT DesA F	CCACCGGCGGCACCACCGACTT	SCO2782 (desA)
RT DesA R	CAGGGAGGCGAGCAGCCCGCAG	
RT cchE F	CCCGGGGCGTCACCGTCGGGTA	SCO0495 (cchE)
RT cchE R	GGGTCCGGAGCGCGGCGATGTC	
RT 7400 F	TGGTGGGCCCGAACGGCAGTGG	SCO7400
RT 7400 R	CGGTGGCGCCGTCCAGGCTGAG	
RT SodF F	AGGGCGCCAACGACACGCTGGA	SCO2633 (sodF)
RT SodF R	CCAGCTCGCCCACGCCGTCCTT	

[a]: F and R refer to the forward and reverse of the coding region; UP and DW refer to the upstream and downstream of the flanking regions for each gene.

Reference

1. Flett, F., Mersinias, V., & Smith, C. P. (1997). High efficiency intergeneric conjugal transfer of plasmid DNA from *Escherichia coli* to methyl DNA-restricting streptomycetes. *FEMS microbiology letters*, *155*(2), 223-229.

2. Bierman, M., Logan, R., O'brien, K., Seno, E. T., Rao, R. N., & Schoner, B. E. (1992). Plasmid cloning vectors for the conjugal transfer of DNA from *Escherichia coli* to *Streptomyces* spp. *Gene*, *116*(1), 43-49.
